# Astrochemical Significance of C_2_H_7_NO Isomers: A Computational Perspective on Their Stability and Detectability

**DOI:** 10.1021/acs.jpca.5c01086

**Published:** 2025-05-15

**Authors:** Lisset Noriega, Luis Armando Gonzalez-Ortiz, Filiberto Ortíz-Chi, Gabriel Merino

**Affiliations:** † Departamento de Física Aplicada, Centro de Investigación y de Estudios Avanzados, 130299 Unidad Mérida, km 6 Antigua Carretera a Progreso, Apdo. Postal 73, Cordemex 97310 Mérida, Yucatán, Mexico; ‡ Secihti-Departamento de Física Aplicada, Centro de Investigación y de Estudios Avanzados, Unidad Mérida, km 6 Antigua Carretera a Progreso, Apdo. Postal 73, Cordemex 97310 Mérida, Yucatán, Mexico

## Abstract

Nitrogen- and oxygen-containing molecules play a key role in interstellar chemistry, particularly as precursors to biologically relevant species such as amino acids. Among the C_2_H_7_NO isomers, 2-aminoethanol is the only one detected in the ISM. This study systematically explores the C_2_H_7_NO chemical space, identifying eight structural isomers, with 1-aminoethanol as the global minimum and methylaminomethanol, 11.5 kcal/mol higher in energy, as a viable higher-energy species. To assess their astrochemical relevance, we conducted a comprehensive conformational analysis and computed rotational constants to guide future spectroscopic searches. These findings provide critical insights into C_2_H_7_NO isomers, identifying new candidates for ISM detection and expanding our understanding of nitrogen- and oxygen-containing organic species in space.

## Graphical abstract

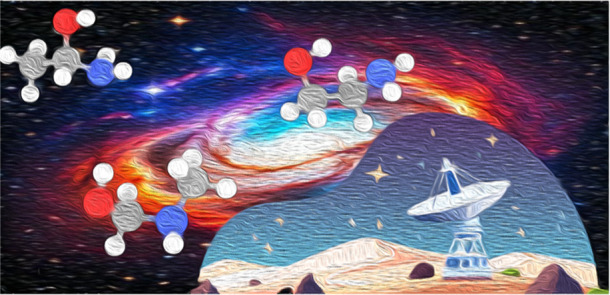

## Introduction

The discovery of complex organic molecules (COMs) in the interstellar medium (ISM) is crucial for understanding chemical processes that may have contributed to the origin of life on Earth.^1,2^ Among the approximately 320 species identified in the ISM, about one-third are COMs, defined as molecules with six or more atoms.^3^ Advances in radio astronomical spectrometry^4,5^ have significantly expanded COMs detection across various ISM environments, from cold dark clouds (around 10 K) to warmer prestellar regions (up to 300 K).^6,7^ Notably, Sagittarius B2 (Sgr B2), one of the most active star-forming regions, hosts nearly one-third of these identified COMs.^3,8,9^

Particular attention has been given to nitrogen- and oxygen-bearing COMs, especially those with peptide bonds [−NH–C­(O)−], due to their potential role as intermediates in amino acid formation, the building blocks of life.^10–12^ Glycine, the simplest amino acid, has been detected in meteorites like Murchison,^13,14^ comets such as 67P/Churyumov-Gerasimenko,^15^ and interstellar ice-analogs,^16,17^ suggesting its possible presence in the ISM. However, despite extensive efforts, glycine has not been definitively identified in astronomical sources.^18^

Several potential glycine precursors, including methylamine (CH_3_NH_2_), formamide (CH_3_CHO), aminoacetronitrile (NH_2_CH_2_CN), and ethanolamine (NH_2_CH_2_CH_2_OH), have been detected in Sgr B2.^3,19,20^ Ethanolamine, the only C_2_H_7_NO isomer observed in the ISM, has also been found in the Almahata Sitta meteorite^21^ and synthesized under laboratory conditions in interstellar ice analogs.^22^ Another isomer, 1-aminoethanol, has been proposed as a molecule of astronomical interest but remains undetected in the ISM.^23,24^

Marloie et al.^23^ suggested that 1-aminoethanol, a chiral molecule, could be identified in the ISM based on the principle of minimum energy,^25^ which establishes that the most stable isomer is likely the most abundant. However, recent studies show that higher-energy isomers can also be prevalent in the ISM, as seen in systems like C_4_H_3_N,^26^ C_3_H_4_O,^27,28^ C_3_H_8_O_2_,^29^ and C_2_H_2_N_2_.^30^ This raises key questions: are ethanolamine and 1-aminoethanol the only relevant isomers of C_2_H_7_NO in astrochemistry? Could other isomers also play a significant role?

To address this, we systematically explored the potential energy surface (PES) of C_2_H_7_NO to better understand its chemical diversity and potential astronomical relevance. We conducted a thorough conformational analysis of all isomers, identifying low-energy structures as promising candidates for astronomical detection, particularly those related to ethanolamine, the only C_2_H_7_NO isomer detected in the ISM to date. Interestingly, several features of the C_2_H_7_NO PES, including energy ordering and molecular geometries, closely resemble those of C_2_H_6_O_2_.^31^

## Methodology

We began by generating all possible constitutional isomers for the molecular formula C_2_H_7_NO. While manual sketching is feasible for small systems, it becomes impractical as molecular complexity increases. To streamline this process, we used SMILES notation, an efficient method for representing chemical structures. Although C_2_H_7_NO is relatively simple, it served as a test case to evaluate the scalability of this approach for more complex molecules.

Given the index of hydrogen deficiency (IHD) of zero, we generated C_2_H_7_NO isomers by starting with SMILES strings for C_4_H_10_ (e.g., CCCC and CC­(C)­C) and substituting two carbon atoms with one oxygen and one nitrogen atom. After removing redundant cases, we identified eight distinct isomers coded in SMILES: CCNO, CCON, CNCO, CNOC, COCN, NCCO, CC­(N)­O, and CN­(C)­O. This set included one chiral molecule; however, only one enantiomer was considered due to their identical energy levels and rotational constants.

We then performed a conformational search for each constitutional isomer. Initial optimizations were performed at the M06-2X-D3/aug-cc-pVTZ level,^32,33^ incorporating Grimme’s D3 dispersion correction.^34^ The optimized structures were refined at the MP2/aug-cc-pVTZ level,^35^ with final energy refinements carried out at CCSD­(T)/aug-cc-pVTZ//MP2/aug-cc-pVTZ level.

The Boltzmann distribution was applied to the conformers using the following equation

$$P\left(E_{i}\right)=\frac{g_{i}e^{-\frac{{\Delta E}_{i}}{kT}}}{Z}$$

where *P*(*E*_*i*_) is the probability of occupying the state with energy *E*_*i*_, *g*_*i*_ is the multiplicity (or degeneracy) of the state *i*, Δ*E*_*i*_ is the relative energy of conformer *i*, *k* is the Boltzmann constant, *T* is the temperature in Kelvin (K), and *Z* is the partition function whose sum is given over all *i* states as

$$Z=\sum g_{i}e^{-\frac{{\Delta E}_{i}}{kT}}$$

Ground-state rotational constants were calculated by incorporating rotational–vibrational coupling at the vibrational perturbation theory to second order (VPT2) level,^36^ along with anharmonic and quartic centrifugal distortion corrections at the MP2/aug-cc-pVTZ level of theory. Conformational searches were carried out using GLOMOS,^37^ and all quantum chemical calculations were performed with Gaussian 16.^38^

## Results and Discussion

The eight structural isomers of C_2_H_7_NO span an energy range of 49.1 kcal/mol (Figure 1). This family includes one chiral molecule, 1-aminoethanol (**1**), which is the most stable structure. The second most stable isomer lies 7.6 kcal/mol above the global minimum and is the only C_2_H_7_NO isomer detected in the ISM so far.^20^ Isomers **1** to **4** are within 14.0 kcal/mol, while isomers **5** to **8**, which feature O–N bonds characteristic of hydroxylamine derivatives, range from 38.5 to 49.1 kcal/mol.

**Figure 1:**
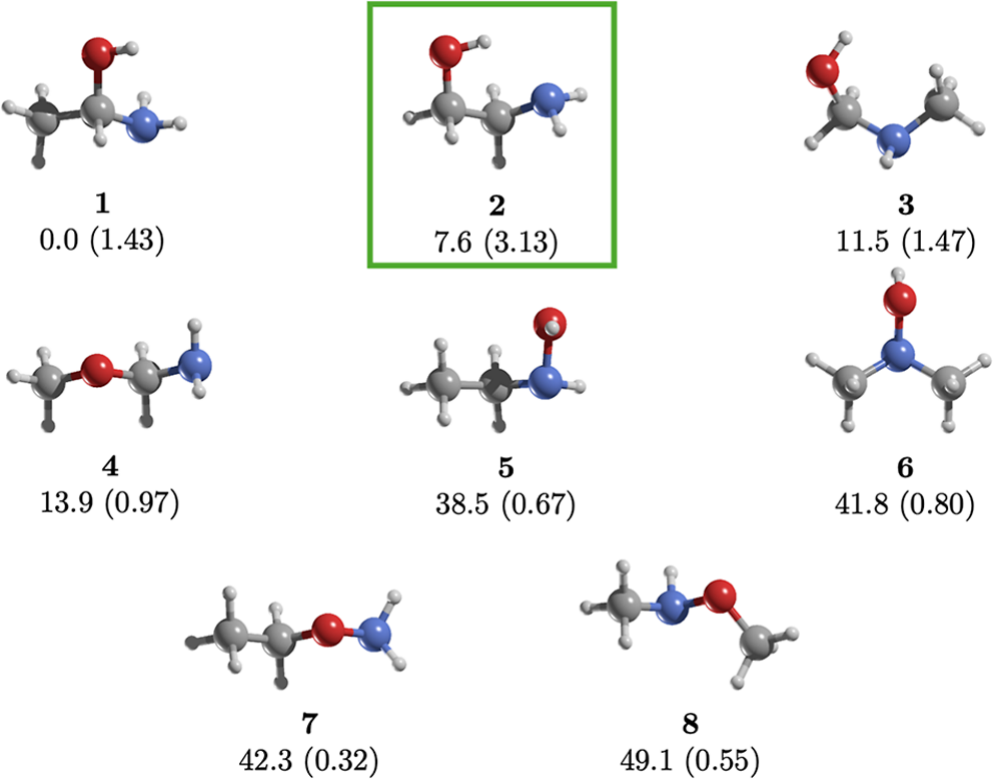
MP2/aug-cc-pVTZ geometries of the most energetically stable conformer of each of the C_2_H_7_NO structural isomers. Relative energies (kcal/mol) are calculated at the CCSD­(T)/aug-cc-pVTZ//MP2/aug-cc-pVTZ level. Dipole moments (in parentheses) are in Debye. Isomer **2** (highlighted with a square) is the only one detected in the ISM.

### 1-Aminoethanol (**1**, Global Minimum)

The most stable isomer, 1-aminoethanol (NH_2_CH­(OH)­CH_3_), features hydroxyl and amino groups on the same carbon atom. It exhibits seven different conformers within a 4.0 kcal/mol range. To determine the most populated conformers under conditions relevant to nebulae and star-forming regions, we calculated Boltzmann distributions across temperatures from 10 to 298 K.^7^

Figure 2 shows the geometries and Boltzmann distribution of these conformers. The three most stable conformers, all within 0.1 kcal/mol of each other, dominate the Boltzmann probabilities at 298 K. As the temperature decreases, the contribution shifts predominantly toward conformer **1-1**, which has a dipole moment of 1.43 D. This suggests that at low temperatures, the detection of conformer **1-1** should be enhanced.

**Figure 2:**
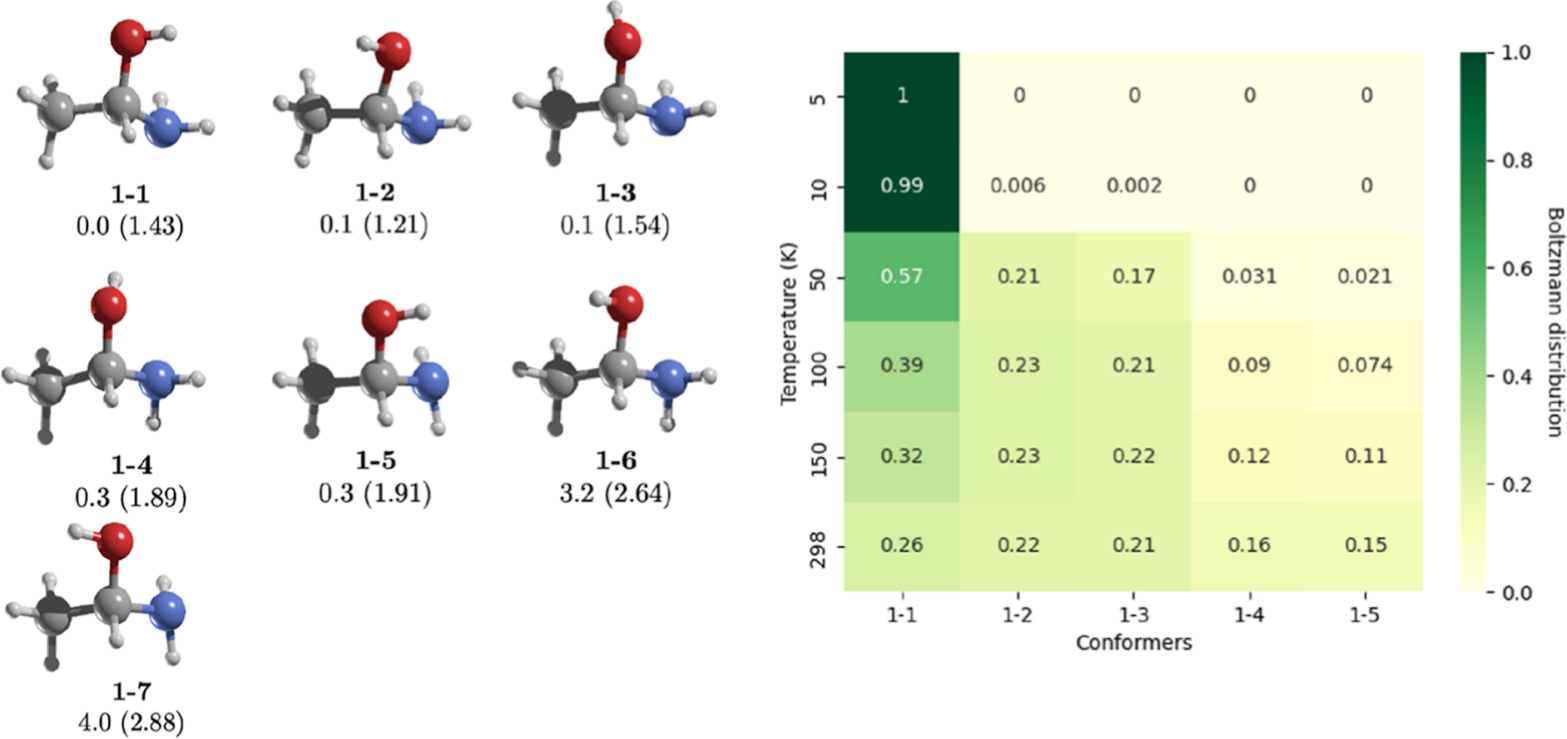
On the left, MP2/aug-cc-pVTZ geometries of the conformers of **1**. Relative energies (kcal/mol) were calculated at the CCSD­(T)/aug-cc-pVTZ//MP2/aug-cc-pVTZ level, and dipole moments (in parentheses) are in Debye. On the right, the heat map shows Boltzmann distribution across temperatures (K), with darker green indicating higher probabilities and lighter shades indicating lower ones. Conformers **1-6** and **1-7**, which have negligible contribution, are omitted for clarity.

The formation of **1** under tropospheric conditions has been studied through both quantum chemical calculations and experimental studies. Initial computational work on the ammonolysis of acetaldehyde showed a barrier of 32.8 kcal/mol. Catalysis by water or formic acid lowers this barrier to 18.7 and 14.0 kcal/mol, respectively.^39^ Nevertheless, these values remain high for standard interstellar conditions.

More pertinent to astrochemical contexts, recent experiments using interstellar ice analogs of NH_3_ and CH_3_CHO showed the formation of **1** at temperatures as low as 65 K.^40^ When one ammonia molecule was explicitly included in the computational model, the barrier decreased to 6.7 kcal/mol. Experimentally, product formation increased sharply above 110 K, suggesting a crossover from quantum tunneling at low temperatures to thermally activated mechanisms. However, even this temperature is insufficient to overcome the reduced barrier via classical Arrhenius behavior, implying that tunneling plays a key role. The discrepancy between theoretical and experimental results may arise from modeling limitations, such a restricted microsolvation and the use of implicit solvents, which tend to overestimate barriers and underestimate tunneling contributions.^40^

Further astrochemical relevance is provided by Duvernay et al.,^41^ who reported the formation of **1** in water-ice at 120 K after 1 h of reaction. They determined an activation energy of 7.9 ± 0.5 kcal/mol and a pre-exponential factor of (7 ± 1) × 10^10^ s^–1^. Under a photon flux typical of shielded dense cloud regions (∼10^4^ photons cm^–2^ s^–1^), isomer **1** reaches peak abundance (∼1% relative to H_2_O) within one month to 10^5^ years at 90–110 K, and within 1000 years at 130 K, temperatures attainable in warm interstellar environments such as hot cores, protoplanetary disks, and cometary nuclei.

In contrast, under stronger VUV radiation (∼10^8^ photons cm^–2^ s^–1^), representative of the diffuse interstellar medium, isomer **1** undergoes rapid photodissociation and becomes undetectable within 1000 years, regardless of temperature. These findings suggest that warm, UV-shielded regions (*T* > 80 K) are the most favorable for its solid-phase detection. If isomer **1** survives desorption, it may also be observable in the gas phase through its rotational transitions in hot core regions.^41^

At these temperatures, the Boltzmann distribution includes several species (**1-1**, **1-2**, and **1-3**), potentially complicating detection due to spectral congestion. This contrasts with isomer **2** (Figures 3 and 4), where conformer **2-1** predominates and was successfully detected in 2021. Structurally, isomer **1** resembles aminomethane (NH_2_CH_2_OH), a known intermediate in glycine synthesis,^42–44^ and may contribute to the formation of amino acids such as alanine.^41^ Therefore, despite the challenges, isomer **1** remains a compelling candidate for the detection of complex organic molecules in the interstellar medium, with potential implications for the origins of biologically relevant compounds.

**Figure 3:**
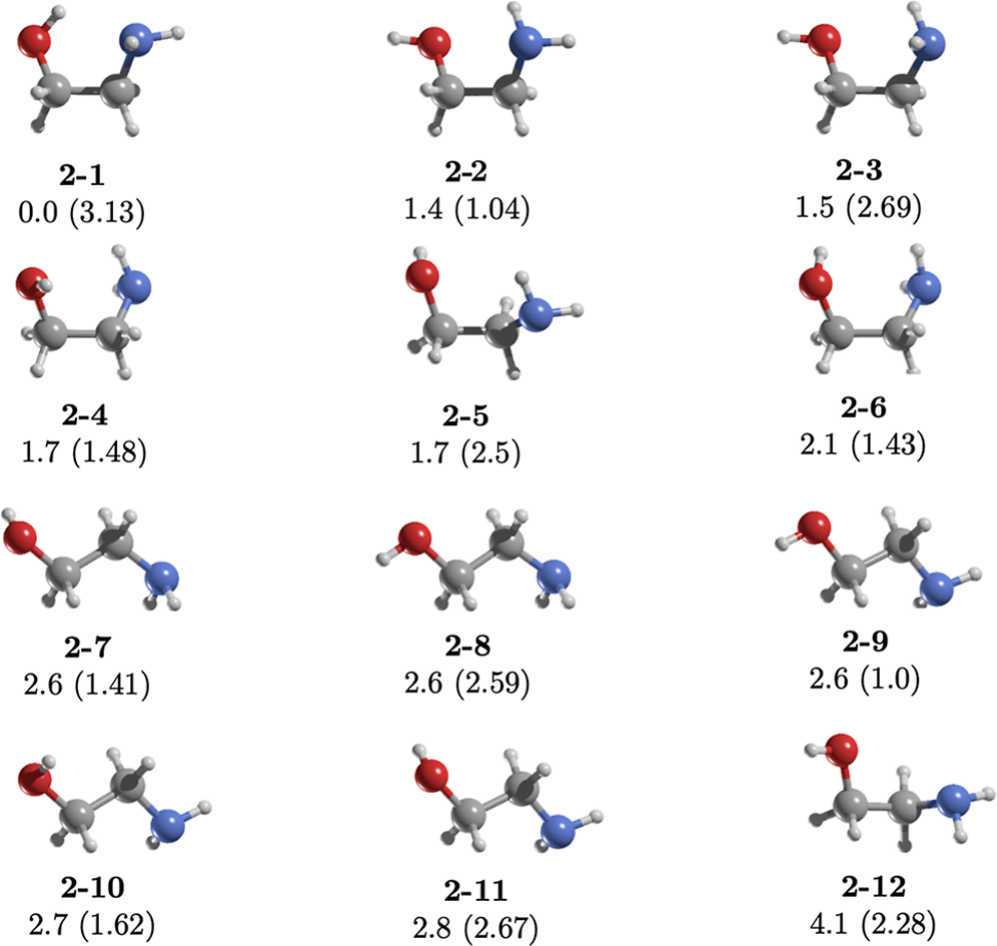
MP2/aug-cc-pVTZ geometries of the conformers of **2**. Relative energies (kcal/mol) were calculated at the CCSD­(T)/aug-cc-pVTZ//MP2/aug-cc-pVTZ level. Dipole moments (in parentheses) are in Debye.

**Figure 4:**
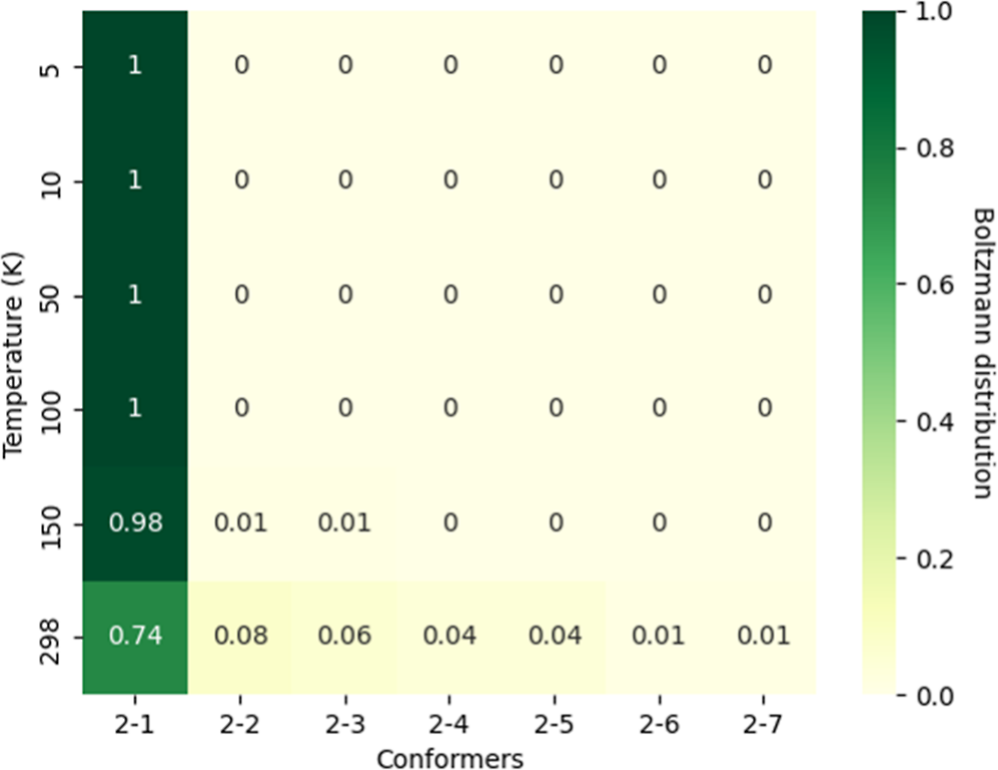
Heat map of the Boltzmann distribution across temperatures (K), with darker green indicating higher probabilities and lighter shades indicating lower ones. Conformers **2-8** to **2-12**, which have negligible distribution, are omitted for clarity.

### 2-Aminoethanol (**2**)

Also known as ethanolamine (NH_2_CH_2_CH_2_OH), this isomer features a hydroxyl group on one carbon atom and an amine group on another. It exhibits 12 conformers within a 4.1 kcal/mol energy range, consistent with previous reports.^45^ The most stable conformer, **2-1**, is stabilized by an intramolecular hydrogen bond (IHB) with an OH···NH distance of 2.2 Å. In comparison, conformer **2-2**, stabilized by an NH···OH IHB (2.4 Å), is 1.4 kcal/mol less stable (Figure 3).

Silva et al.^46^ reported IR and Raman spectra for **2** in both the gas and liquid phases, with theoretical calculations indicating that conformers **2-2** and **2-8** dominate in the liquid phase, while **2-1** prevails in the gas phase. Conversely, Novakovskaya and Rodnikova^47^ proposed **2-11** as the primary conformer in crystalline structures, with **2-2**, **2-3**, and **2-6** likely present in the liquid phase. These findings highlight distinct conformational preferences across gas, liquid, and crystalline phases.

From the Boltzmann distribution analysis, it is evident that at higher temperatures (e.g., 298 K), conformer **2-1** is the dominant species, contributing approximately 74% to the overall ensemble. The remaining conformers contribute between 1 and 8%. As the temperature decreases from 150 to 5 K, conformer **2-1** becomes nearly 100% dominant, indicating that even a moderate energy difference of about 1.0 kcal/mol effectively suppresses the distribution of higher-energy conformers at low temperatures.

In contrast, isomer **1** shows a broader energy distribution, with a more even spread of conformers between 298 and 50 K. This suggests that detecting a unique rotational signature for isomer **1** may be more challenging, especially in cold interstellar environments. Meanwhile, the sharp Boltzmann preference for a single conformer in isomer **2** increases the likelihood of identifying and assigning its spectroscopic features. While Boltzmann distribution alone cannot predict detectability, as factors like conformational interconversion barriers, kinetic accessibility, and formation pathways also play critical roles, this analysis provides valuable insight into the relative observability of different species.

Conformer **2-1** was detected in the ISM in 2021 ^20^ using the rotational spectra from laboratory studies.^48^ Ethanolamine has been proposed as a direct glycine precursor under simulated archean hydrothermal vent conditions^49^ and reacts with formic acid (HCOOH) to produce protonated alanine.^50^ However, its formation mechanisms in the ISM remain unclear. Proposed pathways include hydrogenation of HNCCO on dust grain surfaces^20,51,52^ or surface reaction involving CH_2_OH + NH_2_CH_2_ and NH_2_ + C_2_H_4_OH.^53^ Notably, NH_2_CH_2_ a key intermediate in these reactions-has yet to be detected in the ISM.

### Methylaminomethanol (**3**)

Isomer **3** (CH_3_NHCH_2_OH) features the N–C–O unit and adopts nine conformers within a 3.9 kcal/mol energy range, distinguished by variation in the H–O–C–N (X) and O–C–N–C (Y) dihedral angles. Following ref. 54 notation, these angles are labeled as *XY* (*X*, *Y* = *G* ≈ 60°, *G*′ ≈ −60°, or *T* ≈ 180°). We identified **3-1** (*GG*′) as the most stable conformer, contrasting a prior report that proposed **3-3** (*GG*) as the lowest-energy structure.^54^ The two lowest-energy conformers differ only in OH group rotation, both exhibiting an O–H···N distance of 2.7 Å. However, the N–H···O distance is shorter in **3-1** (2.7 vs 2.8 Å in **3-2**), contributing to its slight stability. Conformers **3-1**, **3-2**, and **3-3** exhibit dipole moments of 1.47, 1.60, and 1.96 D, respectively. These conformers are energetically close, differing by 0.3 kcal/mol. They show a significant probability at 100–150 K (12–56%, Figure 5).

**Figure 5:**
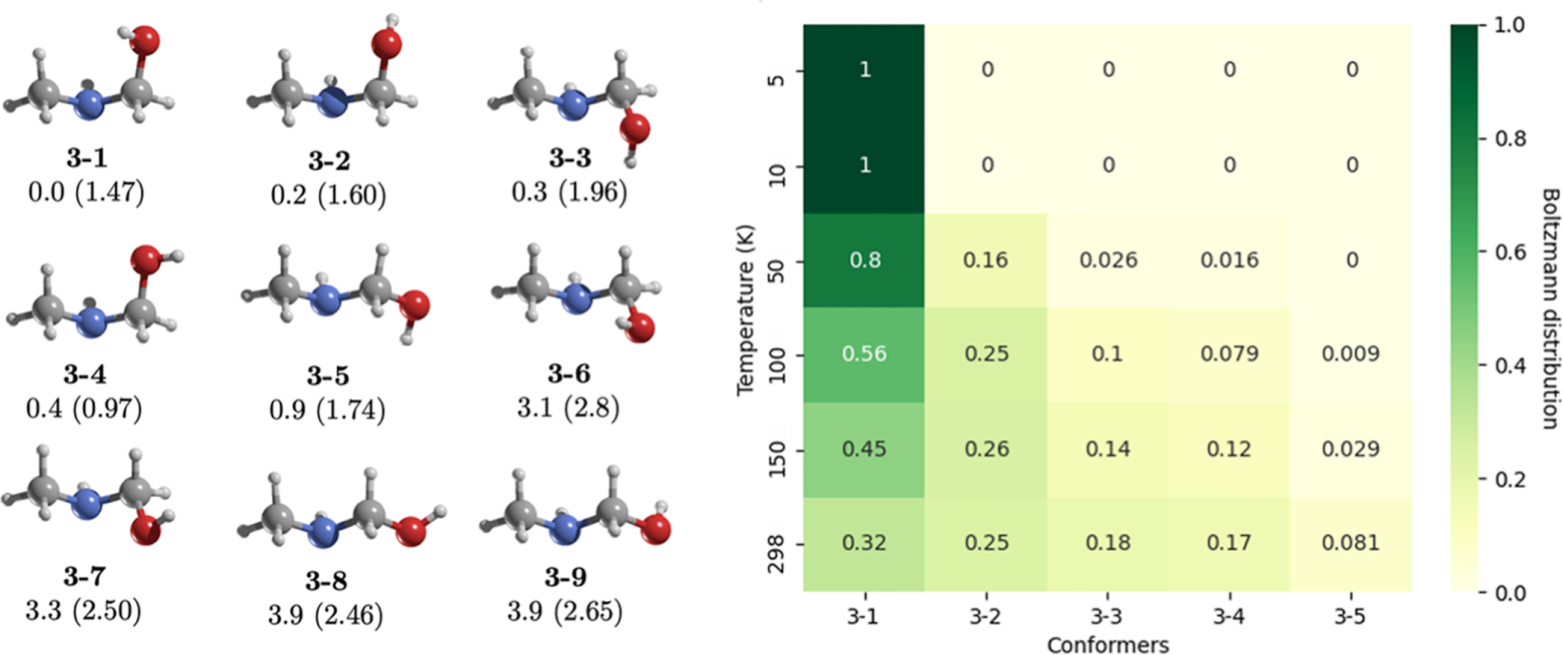
On the left, MP2/aug-cc-pVTZ geometries of the conformers of **3**. Relative energies (kcal/mol) were calculated at the CCSD­(T)/aug-cc-pVTZ//MP2/aug-cc-pVTZ level, and dipole moments (in parentheses) are in Debye. On the right, the heat map shows the Boltzmann distribution across temperatures (K), with darker green indicating higher probabilities and lighter shades indicating lower ones. Conformers **3-6** to **3-9,** which have negligible contribution, are omitted for clarity.

Isomer **3** is a promising candidate for ISM detection, lying 11.5 kcal/mol above the global minimum and 3.9 kcal/mol above the detected isomer **2**. Furthermore, experimental studies indicate that methylamine and formaldehyde react readily in water ice analogs at astronomically relevant temperatures to form isomer **3**, with an experimentally derived activation energy of only 0.024 kcal/mol.^55^ These findings strongly suggest that isomer **3** could exist in the ISM and warrants investigation in future observational studies.

### Methoxymethanamine (**4**)

Isomer **4** contains the O–C–N unit and its stability is attributed to the anomeric effect.^54^ It adopts four conformers within a 3.8 kcal/mol energy range (Figure 6). The two most stable conformers, **4-1** (*TT*) and **4-2 (** *G*′*T*), differ in methyl group orientation (C–O–C–N dihedral angles: −180.0° vs −70.3°), though both retain an N lone pair (Lp)–N–C–O dihedral angle of 180.0°. Consistent with ref 54, **4-2** displays a stronger dipole moment (1.46 vs 0.96 D for **4-1**), making it more detectable via rotational microwave spectroscopy. Given its structural features and dipole moment, isomer **4** emerges as an intriguing candidate for further astrochemical studies, particularly in the context of rotational spectroscopy and potential formation pathways under interstellar conditions.

**Figure 6:**
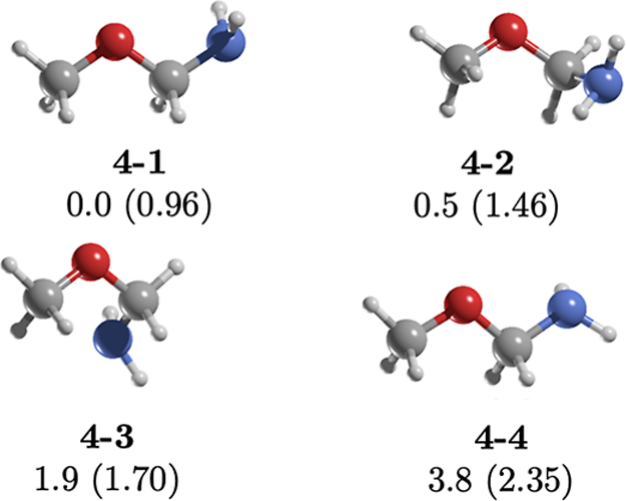
MP2/aug-cc-pVTZ geometries of the conformers of **4.** Relative energies (kcal/mol) were calculated at the CCSD­(T)/aug-cc-pVTZ//MP2/aug-cc-pVTZ level. Dipole moments (in parentheses) are in Debye.

### Hydroxylamine Derivatives (**5**–**8**)

Hydroxylamine (NH_2_OH), a prebiotic precursor for amino acids and pyrimidine ribonucleotides,^57^ was recently detected in the ISM toward *G* + 0.693 – 0.027 in Sgr B2.^58^ Several studies have aimed to elucidate its formation mechanism and the factors behind its low abundance in this region.^59,60^ We identified four hydroxylamine derivatives (**5**-**8**) at a range energy of 38.5–49.1 kcal/mol. All contain the unstable N–O bond, likely explaining their elevated energies.^61^

*N*-Ethylhydroxylamine (**5**), CH_3_CH_2_NHOH, formed by replacing a hydrogen on NH_2_OH’s nitrogen with an ethyl group, adopts six conformers. The three most stable (**5**-**1** to **5-3**, 0.0–0.4 kcal/mol apart) exhibit dipole moments below 0.90 D and share an H–O–N–C dihedral angle of ∼125.6°. Their O–N–C–C dihedral angle (−75.4, 170.2, and 56.6°) classify them as *GG*′, *GT*, and *GG*, respectively.

CH_3_N­(OH)­CH_3_ (41.8 kcal/mol above **1**) replaces two hydrogens on NH_2_OH’s nitrogen with methyl groups. The most stable conformer, **6-1**, features a shorter N–O distance (1.45 vs 1.43 Å in **6-2**) and a stronger O–H···N interaction (1.90 vs 1.94 Å), enhancing stability by 2.3 kcal/mol.

CH_3_CH_2_ONH_2_ (42.3 kcal/mol above **1**) substitutes the OH hydrogen in NH_2_OH with an ethyl group. Its four conformers span a 3.3 kcal/mol range, with the two lowest-energy structures (**7-1** and **7-2**) differing in C–C–O–N dihedral angles (180.0° vs 70.6°) and dipole moments <0.48 D.

Finally, CH_3_NHOCH_3_ (49.1 kcal/mol above **1**), the highest-energy isomer, replaces NH_2_OH’s OH hydrogen and one NH_2_ hydrogen with methyl groups. Its two conformers, differing by 3.8 kcal/mol, arise from rotation around the O–C bond. Hydroxylamine derivatives are known as reducing agents or organic reaction intermediates, with reactivity centered on the NHO moiety.^62–64^ While their astrochemical relevance remains unexplored, studying their stability and formation pathways could advance our understanding of interstellar chemistry. The conformers and their Boltzmann distribution can be consulted in the Supporting Information, Figures S1 and S2, as well as Table S1.

### Boltzmann-averaged Dipole Moments

To assess detectability under interstellar conditions, we analyzed the Boltzmann-averaged dipole moments of each isomer as a function of temperature (Figure S3). Although isomer **1** is the global minimum in energy, isomer **2** shows the highest dipole moment at low temperatures, reinforcing that strong dipole moments favor astronomical detection.

In general, dipole moments increase with temperature due to the thermal population of more polar, higher-energy conformers. However, isomer **2** deviates from this trend: its averaged dipole moment decreases at 298 K, because its higher-energy conformers have lower dipole moments than the dominant low-energy conformer **2-1** (Figure 3).

Despite their higher relative energies, isomers **6**-**8** exhibit moderate dipole moments across the temperature range. This indicates that dipole strength alone is not sufficient for detectability; energy, abundance, and formation pathways must also be taken into account. Nonetheless, our results support prioritizing low-energy, high-dipole species such as isomers **1**-**3** for future ISM searches.

### Parallelism between the PES Exploration of C_2_H_6_O_2_ and C_2_H_7_NO

A striking parallel emerges when comparing the energy landscapes of C_2_H_7_NO and C_2_H_6_O_2_.^31^ The interchangeability of nitrogen and oxygen in C_2_H_7_NO results in an energy hierarchy that closely mirrors that of C_2_H_6_O_2_ (Figure 7). This resemblance is not coincidental but rather indicative of a broader pattern in molecular stability.

**Figure 7:**
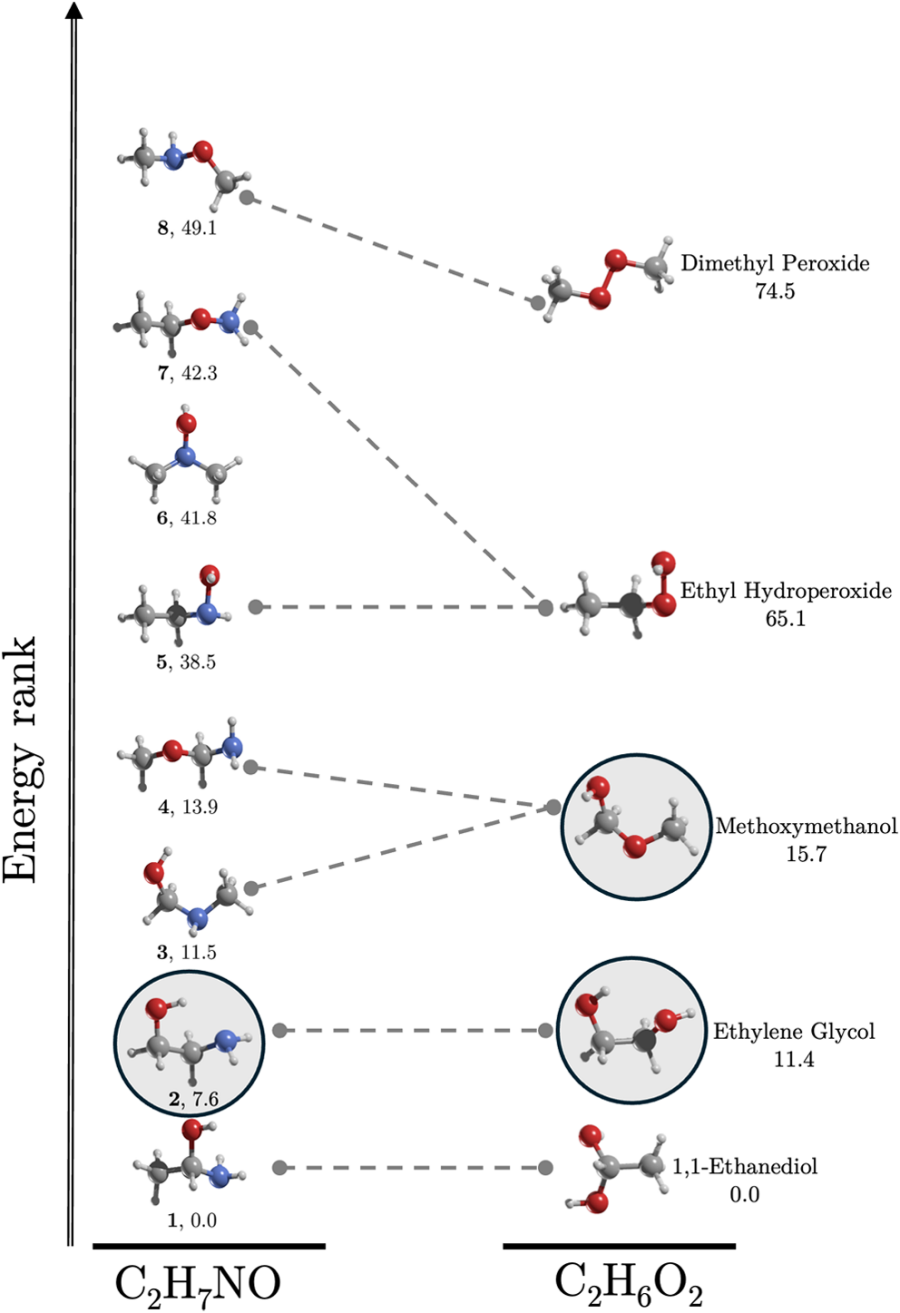
Comparison of the energy rankings of C_2_H_7_NO and C_2_H_6_O_2_ isomers at the CCSD­(T)/aug-cc-pVTZ//MP2/aug-cc-pVTZ level. Isomers enclosed in circles have been detected in the ISM.

At the core of this parallel lies isomer **1**, the most stable form of C_2_H_7_NO, which directly corresponds to 1,1-ethanediol, the most stable isomer of C_2_H_6_O_2_. Similarly, isomer **2**, structurally aligned with ethylene glycol, holds the second position in stability in both systems. The trend continues with isomers **3** and **4**, which mirror the energetic placement of methoxymethanol, the third most stable species in C_2_H_6_O_2_. Further down the stability ladder, isomers **5** and **7** resemble ethyl hydroperoxide, while isomer **8**, the least stable C_2_H_7_NO species, corresponds to dimethyl peroxide, the highest-energy isomer in C_2_H_6_O_2_.

This structural and energetic alignment raises intriguing questions about the potential presence of these C_2_H_7_NO isomers in the ISM. Ethylene glycol, the second-most stable species in C_2_H_6_O_2_, has already been detected in the ISM, along with methoxymethanol. Given their close analogies, could isomers **3** and **4** of C_2_H_7_NO also exist in space?

While direct astronomical evidence remains elusive, the observed trend justifies further exploration. Laboratory spectroscopy, combined with high-sensitivity radioastronomical surveys, could guide future searches by providing essential spectroscopic data and evaluating their observational feasibility. Additionally, investigating their formation pathways under ISM-like conditions may shed light on their potential role as interstellar species. If these isomers indeed follow the molecular stability patterns observed in C_2_H_6_O_2_, they may play an unexplored role in interstellar N–O chemistry, warranting deeper theoretical and experimental investigation.

### Rotational Constants

Most molecules identified in the ISM have been detected through rotational spectroscopy, which requires rotational transitions and a nonzero dipole moment.^65^ Rotational constants are highly sensitive to small geometric variations caused by vibrations. While the rigid rotor approximation within the harmonic oscillator framework provides equilibrium rotational constants, ground-state rotational constants—more relevant under the low-temperature conditions of the ISM—are significantly affected by anharmonicity.^66^ Therefore, anharmonic corrections must be applied, as they influence molecular vibrations, moments of inertia, and rotational constants.

At the VPT2 level, anharmonic corrections result in deviations of 0.65%, 0.13%, and 0.04% for *A*_0_, *B*_0_, and *C*_0_, respectively, compared to experimental values for isomer **2-1**, the only isomer with available experimental data (Table S2).^48^ These small deviations confirm the reliability of our computational approach in predicting vibrationally corrected rotational constants.

Table 1 summarizes the vibrationally corrected rotational constants for the most stable conformers of the structural isomers, while the complete set of conformers for all isomers is available in Table S3 of the Supporting Information. All conformers are asymmetric tops (*A*_0_ > *B*_0_ > *C*_0_) and possess nonzero dipole moments, confirming their rotational activity. Notably, isomers **1**, **2**, and **3** exhibit the largest dipole moments among the studied species, enhancing their detectability in the ISM and making them prime candidates for further spectroscopic characterization.

**Table 1:** Vibrationally Corrected Rotational Constants (*A*_0_, *B*_0_, *C*_0_, MHz), Dipole Moment Components (μ_a_, μ_b_, μ_c_, Debye), and Total Dipole Moment (μ, Debye) at the MP2/aug-cc-pVTZ Level for the Lowest-Energy conformer of the C_2_H_7_NO Structural Isomers

Conf	Δ*E*	*A*_0_	*B*_0_	*C*_0_	μ_a_	μ_b_	μ_c_	|μ|
**1-1**	0.0	8832.84	8471.59	4927.52	1.27	0.45	0.48	1.43
**2-1**	7.6	14414.19	5553.83	4568.46	–2.86	1.17	0.55	3.13
**3-1**	11.5	15826.24	5255.70	4487.35	–1.05	0.91	0.49	1.47
**4-1**	13.9	29970.96	4223.579	3969.984	–0.93	0.26	0.00	0.96
**5-1**	38.5	15153.49	5273.2	4473.245	–0.42	0.48	–0.19	0.67
**6-1**	41.8	9136.696	8992.886	5152.727	0.00	–0.65	–0.46	0.80
**7-1**	42.3	29686.8	4122.006	3870.311	0.32	–0.05	0.00	0.33
**8-1**	49.1	22591.82	4557.302	4494.632	0.50	–0.13	–0.19	0.55

## Conclusion

This study provides a comprehensive analysis of the C_2_H_7_NO chemical space, identifying eight structural isomers with varying energetic stabilities using a SMILES-based exploration. Among them, 1-aminoethanol (the global minimum) and ethanolamine are of particular astrochemical relevance, with the latter being the only isomer detected in the ISM thus far. Our findings highlight the potential for other isomers, such as methylaminomethanol, to play a significant role in astrochemistry, despite their higher energy states. These isomers could offer new candidates for ISM detection and warrant further experimental investigation.

Moreover, our results encourage further exploration of the C_2_H_7_NO chemical space, suggesting that additional isomers may yet be discovered and contribute to the pool of molecules relevant to the origins of life. Given the energetic proximity of several of these isomers, future observational studies are needed to assess their presence in space, particularly through advanced spectroscopic methods. Their detection would provide crucial insights into the chemical processes underlying the formation of biologically relevant compounds in the ISM, enhancing our understanding of prebiotic chemistry in extraterrestrial environments. Future missions and telescopic surveys could provide the necessary tools to identify these species, shedding light on the complex molecular diversity of the ISM.

## Supplementary Material



## Notes

All conformers of the hydroxylamine derivatives **5** to **8**; Boltzmann-averaged dipole moments (μ) for each isomer as a function of temperature; Boltzmann distribution of the conformers of C_2_H_7_NO at different temperatures (K); vibrationally corrected rotational constants (*A*_0_, *B*_0_, *C*_0_, MHz) of ethanolamine and structurally related molecules; predicted equilibrium rotational constants (*A*_e_, *B*_e_, *C*_e_, MHz), vibrationally corrected rotational constants (*A*_0_, *B*_0_, *C*_0_, MHz), dipole moment components (μ_a_, μ_b_, μ_c_, Debye), dipole moment (|μ|, Debye) of all conformers of C_2_H_7_NO; and Cartesian coordinates of all the conformers identified (PDF)
