# Supplementary material for: Astrochemical Significance of C2H7NO Isomers: A Computational Perspective on Their Stability and Detectability
Source: J Phys Chem A. 2025 May 15;129(21):4715–23. doi: 10.1021/acs.jpca.5c01086 (PMC12128033; doi:10.1021/acs.jpca.5c01086)
Supplement: Supplementary file 1 [file jp5c01086_si_001.pdf]

Supplemental Material
for

Astrochemical Significance of C_2H_7NO Isomers: A Computational Perspective on Their Stability and Detectability

Lisset Noriega,[1*] Luis Armando González-Ortiz,[1] Filiberto Ortíz-Chi,[2] and Gabriel Merino[1]*

[1]Departamento de Física Aplicada, Centro de Investigación y de Estudios Avanzados, Unidad Mérida, km 6 Antigua Carretera a Progreso, Apdo. Postal 73, Cordemex 97310, Mérida, Yucatán, Mexico.

[2] SECIHTI-Departamento de Física Aplicada, Centro de Investigación y de Estudios Avanzados, Unidad Mérida, km 6 Antigua Carretera a Progreso, Apdo. Postal 73, Cordemex 97310, Mérida, Yucatán, Mexico.

*lisset.noriega@cinvestav.mx; gmerino@cinvestav.mx

Table of content

Figure S1. All conformers of the hydroxylamine derivatives **5** and **6**. Relative energy (kcal/mol) obtained at the CCSD(T)/aug-cc-pVTZ//MP2/aug-cc-pvTZ level of theory. The value in parenthesis represents the dipole moment (Debye).

Figure S2. All conformers of the hydroxylamine derivatives **7** and **8**. Relative energy (kcal/mol) obtained at the CCSD(T)/aug-cc-pVTZ//MP2/aug-cc-pvTZ level of theory. The value in parenthesis represents the dipole moment (Debye).

Figure S3. Boltzmann-averaged dipole moments (μ) for each isomer as a function of temperature, weighted using Boltzmann distributions.

Table S1. Boltzmann distribution of the conformers of C_2H_7NO at different temperatures (in Kelvin) considering the relative energy at the CCSD(T)/aug-cc-pVTZ//MP2/aug-cc-pVTZ.

Table S2. Vibrationally corrected rotational constants (A_0, B_0, C_0, MHz) of ethanolamine and structurally related molecules computed at the MP2/aug-cc-pVTZ level of theory. The percentage error between experimental and theoretical values is given in parentheses.

Table S3. Predicted equilibrium rotational constants (A_e, B_e, C_e, MHz), Vibrationally corrected rotational constants (A_0, B_0, C_0, MHz), dipole moment components (μ_a, μ_b, μ_c, in Debye), and dipole moment (|μ|, Debye) at MP2/aug-cc-pVTZ of all conformers of C_2H_7NO and their relative energy (ΔE, in kcal/mol) at CCSD(T)/aug-cc-pVTZ//MP2/aug-cc-pVTZ.

Cartesian coordinates of all the conformers identified. All the structures are optimized at MP2/aug-cc-pVTZ. The relative energy (ΔE, kcal/mol) includes the zero-point energy correction.

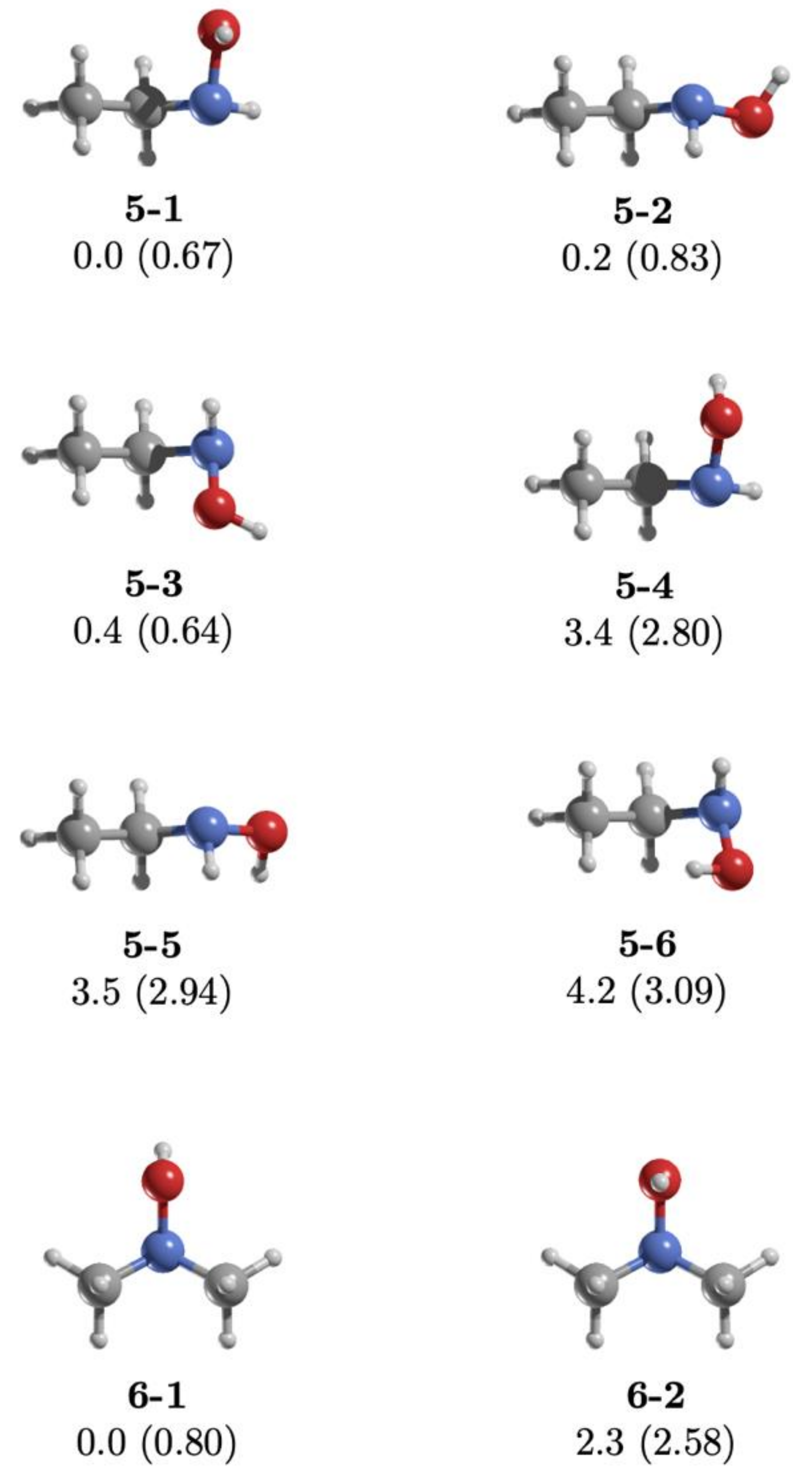

Figure S1. All conformers of the hydroxylamine derivatives **5** and **6**. Relative energy (kcal/mol) obtained at the CCSD(T)/aug-cc-pVTZ//MP2/aug-cc-pvTZ level of theory. The value in parenthesis represents the dipole moment (Debye).

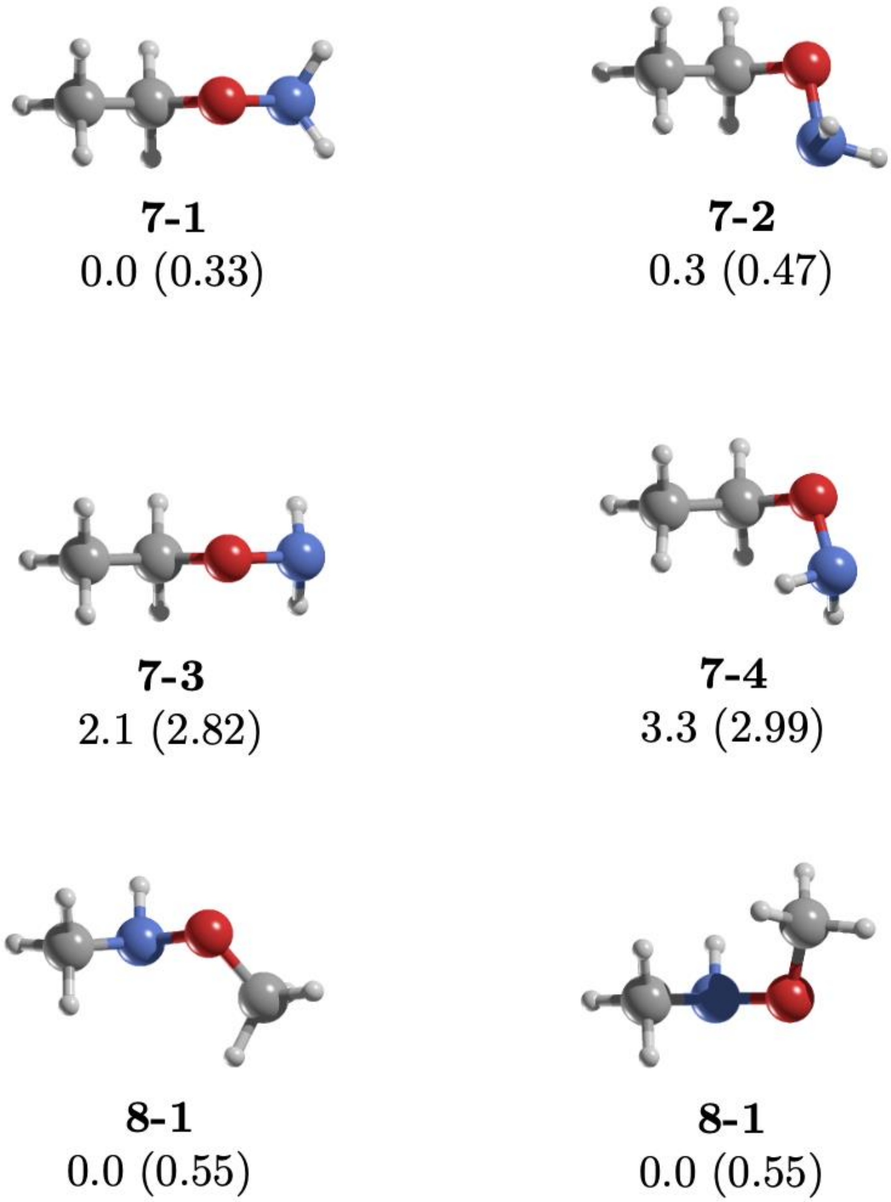

Figure S2. All conformers of the hydroxylamine derivatives **7** and **8**. Relative energy (kcal/mol) obtained at the CCSD(T)/aug-cc-pVTZ//MP2/aug-cc-pvTZ level of theory. The value in parenthesis represents the dipole moment (Debye).

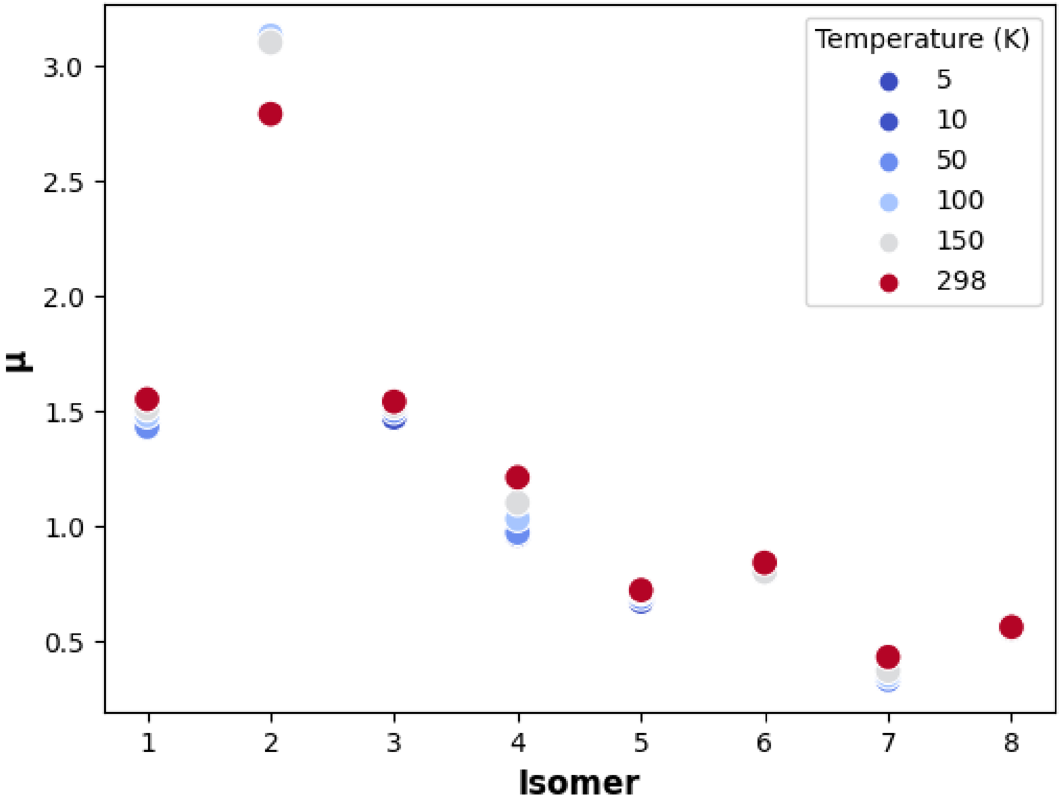

Figure S3. Boltzmann-averaged dipole moments (μ) for each isomer as a function of temperature, weighted using Boltzmann distributions.

Table S1. Boltzmann distribution of the conformers of C_2H_7NO at different temperatures (in Kelvin) considering the relative energy at the CCSD(T)/aug-cc-pVTZ//MP2/aug-cc-pVTZ.

Isomer	5K	10K	50K	100K	150K	298K
1-1	100.0%	99.1%	57.0%	38.9%	32.3%	26.0%
1-2	0.0%	0.6%	20.8%	23.5%	23.1%	21.9%
1-3	0.0%	0.2%	17.0%	21.2%	21.6%	21.2%
1-4	0.0%	0.0%	3.1%	9.0%	12.2%	15.9%
1-5	0.0%	0.0%	2.1%	7.4%	10.7%	14.9%
1-6	0.0%	0.0%	0.0%	0.0%	0.0%	0.1%
1-7	0.0%	0.0%	0.0%	0.0%	0.0%	0.0%
2-1	100.0%	100.0%	100.0%	99.8%	97.6%	73.9%
2-2	0.0%	0.0%	0.0%	0.1%	1.1%	7.6%
2-3	0.0%	0.0%	0.0%	0.0%	0.6%	5.7%
2-4	0.0%	0.0%	0.0%	0.0%	0.3%	4.2%
2-5	0.0%	0.0%	0.0%	0.0%	0.3%	3.9%
2-6	0.0%	0.0%	0.0%	0.0%	0.1%	1.0%
2-7	0.0%	0.0%	0.0%	0.0%	0.0%	1.0%
2-8	0.0%	0.0%	0.0%	0.0%	0.0%	0.4%
2-9	0.0%	0.0%	0.0%	0.0%	0.0%	0.8%
2-10	0.0%	0.0%	0.0%	0.0%	0.0%	0.7%
2-11	0.0%	0.0%	0.0%	0.0%	0.0%	0.7%
2-12	0.0%	0.0%	0.0%	0.0%	0.0%	0.1%
3-1	1.000	1.000	0.798	0.560	0.447	0.322
3-2	0.000	0.000	0.160	0.251	0.261	0.246
3-3	0.000	0.000	0.026	0.101	0.143	0.181
3-4	0.000	0.000	0.016	0.079	0.121	0.167
3-5	0.000	0.000	0.000	0.009	0.029	0.081
3-6	0.000	0.000	0.000	0.000	0.000	0.002
3-7	0.000	0.000	0.000	0.000	0.000	0.001
3-8	0.000	0.000	0.000	0.000	0.000	0.000
3-9	0.000	0.000	0.000	0.000	0.000	0.000
4-1	100.0%	100.0%	98.7%	86.1%	72.6%	51.5%
4-2	0.0%	0.0%	1.3%	13.9%	27.1%	44.3%
4-3	0.0%	0.0%	0.0%	0.0%	0.2%	4.2%
4-4	0.0%	0.0%	0.0%	0.0%	0.0%	0.1%
5-1	100.0%	100.0%	86.1%	65.8%	55.6%	44.4%
5-2	0.0%	0.0%	12.0%	24.5%	28.8%	31.9%
5-3	0.0%	0.0%	1.9%	9.7%	15.5%	23.4%
5-4	0.0%	0.0%	0.0%	0.0%	0.0%	0.1%
5-5	0.0%	0.0%	0.0%	0.0%	0.0%	0.1%
5-6	0.0%	0.0%	0.0%	0.0%	0.0%	0.0%
6-1	100.0%	100.0%	100.0%	100.0%	100.0%	98.0%
6-2	0.0%	0.0%	0.0%	0.0%	0.0%	2.0%
7-1	100.0%	100.0%	95.3%	81.9%	73.2%	61.1%
7-2	0.0%	0.0%	4.7%	18.1%	26.7%	36.9%
7-3	0.0%	0.0%	0.0%	0.0%	0.1%	1.8%
7-4	0.0%	0.0%	0.0%	0.0%	0.0%	0.2%
8-1	100.0%	100.0%	100.0%	100.0%	100.0%	99.8%
8-2	0.0%	0.0%	0.0%	0.0%	0.0%	0.2%

The Boltzmann distribution of the conformers at different temperatures was visualized using a heatmap created with the seaborn, matplotlib, and pandas libraries in Python, using the following code

```
import seaborn as sns
import matplotlib.pyplot as plt
import pandas as pd

# Data
data = {
    'Temperature (K)': ['5', '10', '50', '100', '150', '298'],
     '1-1': [1, 0.991, 0.570, 0.389, 0.323, 0.260],
     '1-2': [0, 0.006, 0.208, 0.235, 0.231, 0.219],
     '1-3': [0, 0.002, 0.170, 0.212, 0.216, 0.212],
     '1-4': [0, 0, 0.031, 0.090, 0.122, 0.159],
     '1-5': [0, 0, 0.021, 0.074, 0.107, 0.149],}

df = pd.DataFrame(data)
df.set_index('Temperature (K)', inplace=True)

# Plot
heatmap = sns.heatmap(df, annot=True, cmap='YlGn')
colorbar = heatmap.collections[0].colorbar
colorbar.set_label('Boltzmann distribution', rotation=270, labelpad=15)
plt.xlabel('Conformers')
plt.show()
```

This dataset corresponds to conformer "**1**" and illustrates the variation in population with temperature. The data was first converted into a pandas DataFrame to facilitate the heatmap generation, enabling clear visualization of conformer populations across the temperature range.

Table S2. Vibrationally corrected rotational constants (A_0, B_0, C_0, MHz) of ethanolamine and structurally related molecules computed at the MP2/aug-cc-pVTZ level of theory. The percentage error between experimental and theoretical values is given in parentheses.

	Exp.	Theor.	Molecular Formula	Ref
		Ethanolamine ($NH_2CH_2CH_2OH$)		
A_0	14508.72725	14414.19 (0.65)		
B_0	5546.49379	5553.83 (0.13)	C_2H_7NO	Widicus, S. L., et al. J. Mol. Spectrosc. **2003**, 217 (2), 278–281.
C_0	4570.48697	4568.46 (0.04)		
		Ethyleneglycol ($OHCH_2CH_2OH$)		
A_0	15214.601	15286.96 (0.48)		
B_0	5538.331	5563.675 (0.46)	$C_2H_6O_2$	Christen, Dines, et al. J. Mol. Spectrosc. 2001, 205(2), 185-196.
C_0	4595.025	4595.464 (0.01)		
		Methoxymethanol (CH_3OCH_2OH)		
A_0	17237.949	17066.64 (0.99)		
B_0	5567.81516	5553.678 (0.25)	$C_2H_6O_2$	Motiyenko, Roman A., et al. Phys. Chem. Chem. Phys.**2018**, 20, 5509-5516.
C_0	4813.04186	4792.068 (0.44)		
		syn-Acrylamide ($CH_2CHC(O)NH_2$)		
A_0	10732.81933	10648.66 (0.78)		
B_0	4218.690256	4238.08 (0.46)	C_3H_5NO	Kolesniková, L., et al. Astron. Astrophys. **2022**, 659, A111
C_0	3030.752979	3020.685 (0.33)		
		Lactonytrile-A ($CH_3CH(OH)CN$)		
A_0	8790.20869	8761.609 (0.33)		
B_0	4005.855754	3982.791 (0.58)	C_3H_5NO	Insausti, A., et al. Astrophys. J. **2025**, 98(1), 64.
C_0	2975.801301	2963.042 (0.43)		
		Lactonitrile-B ($CH_3CH(OH)CN$)		
A_0	8584.18543	8540.517 (0.51)		
B_0	4028.709824	4006.441 (0.55)	C_3H_5NO	Insausti, A., et al. Astrophys. J. **2025**, 98(1), 64.
C_0	2987.844979	2972.276 (0.52)		
		Glycine-I (NH_2CH_2COOH)		
A_0	10341.53	10224.53 (1.13)		
B_0	3876.1798	3864.01 (0.31)	$C_2H_5NO_2$	D. Suenram and F. J. Lovas. J. Am. Chem. Soc. **1980**, 102, 7180
C_0	2912.3525	2896.644 (0.54)		
		Glycine-II (NH_2CH_2COOH)		
A_0	10130.152	10116.37 (0.14)		
B_0	4071.5075	4055.711 (0.39)	$C_2H_5NO_2$	R. D. Suenram and F. J. Lovas. J. Mol. Spectrosc. **1978**, 72, 372
C_0	3007.4892	2994.873 (0.42)		
		Formamide (NH_2CHO)		
A_0	71926.373	72716.954 (1.09)		
B_0	11262.331	11373.453 (0.98)	CH_3NO	W. H. Kirchho, **1972**, J. Mol. Spect. 41, 333.
C_0	9761.643	9833.903 (0.73)		

Table S3. Predicted equilibrium rotational constants (A_e, B_e, C_e, MHz), Vibrationally corrected rotational constants (A_0, B_0, C_0, MHz), dipole moment components (μ_a, μ_b, μ_c, Debye), and dipole moment (|μ|, Debye) at MP2/aug-cc-pVTZ of all conformers of C_2H_7NO and their relative energy (ΔE, in kcal/mol) at CCSD(T)/aug-cc-pVTZ//MP2/aug-cc-pVTZ.

Conf.	ΔE	A_e	B_e	C_e	A_0	B_0	C_0	μ_a	μ_b	μ_c	|μ|
1-1	0.0	8926.07	8554.00	4982.13	8832.84	8471.59	4927.52	1.27	0.45	0.48	1.43
1-2	0.1	8980.16	8527.86	4989.51	8881.17	8446.80	4933.04	-0.92	0.69	0.38	1.21
1-3	0.1	8728.46	8579.13	4976.89	8659.23	8488.28	4921.96	0.37	-0.35	1.45	1.54
1-4	0.3	9052.12	8500.34	4990.86	8951.02	8428.74	4935.94	-0.83	-1.67	0.36	1.89
1-5	0.3	9151.72	8379.26	4992.01	9055.09	8300.42	4937.72	0.25	-1.84	0.42	1.91
1-6	3.2	9088.96	8564.07	4961.09	8996.78	8486.05	4909.86	-1.18	-0.12	2.35	2.64
1-7	4.0	9051.49	8434.46	4980.26	8955.05	8352.89	4925.08	-2.42	-1.54	0.22	2.88
2-1	7.6	14482.04	5661.38	4643.02	14414.19	5553.83	4568.46	-2.86	1.17	0.55	3.13
2-2	8.9	15066.60	5489.75	4559.31	14971.28	5404.11	4493.97	0.62	0.82	-0.13	1.04
2-3	9.1	14955.76	5364.66	4498.03	14833.80	5298.58	4441.68	1.83	1.03	1.67	2.69
2-4	9.3	14836.81	5261.76	4455.34	14736.93	5198.97	4399.57	-0.71	1.27	-0.23	1.48
2-5	9.3	14976.78	5385.35	4517.70	14902.05	5306.33	4455.53	0.28	1.15	2.20	2.50
2-6	9.6	14817.88	5258.44	4395.84	14739.49	5183.80	4345.05	0.23	-0.11	1.41	1.43
2-7	10.1	27896.68	3839.98	3619.65	27585.45	3806.54	3586.34	-0.33	-0.72	1.17	1.41
2-8	10.2	28351.50	3894.41	3644.75	28008.468	3855.404	3609.717	0.69	2.49	0.00	2.59
2-9	10.2	28862.88	3936.26	3665.20	28506.666	3895.25	3628.794	-0.31	0.64	-0.71	1.00
2-10	10.3	28444.56	3883.51	3638.54	28097.008	3848.684	3605.437	1.25	1.01	0.21	1.62
2-11	10.3	28405.88	3885.24	3644.54	28075.237	3849.835	3609.769	-1.35	0.98	2.08	2.67
2-12	11.7	16157.73	4953.34	4392.93	15882.546	4927.622	4349.632	-0.40	1.88	1.22	2.28
3-1	11.5	15861.67	5348.23	4549.93	15826.24	5255.70	4487.35	-1.05	0.91	0.49	1.47
3-2	11.7	15670.93	5369.02	4582.34	15617.74	5290.45	4522.56	0.22	0.42	-1.53	1.60
3-3	11.9	16536.09	5224.83	4581.57	16455.30	5153.40	4518.06	-1.41	1.36	-0.06	1.96
3-4	11.9	15804.73	5476.65	4649.47	15734.78	5381.60	4581.36	-0.78	0.17	0.56	0.97
3-5	12.4	29420.18	4118.03	3862.26	29064.22	4075.29	3823.58	1.74	0.16	-0.01	1.74
3-6	14.7	16618.21	5207.30	4485.46	16719.07	5087.11	4414.48	-1.68	0.08	1.95	2.58
3-7	14.8	16440.78	5321.34	4587.16	16323.01	5254.16	4522.17	-0.31	0.92	2.31	2.50
3-8	15.4	30411.79	4133.32	3854.37	30040.93	4090.40	3816.29	0.46	1.83	1.58	2.46
3-9	15.4	29976.37	4068.08	3823.54	29629.09	4031.91	3790.10	1.13	0.31	2.37	2.65
4-1	13.9	30304.31	4268.56	4010.64	29970.96	4223.579	3969.984	-0.93	0.26	0.00	0.96
4-2	14.4	16584.70	5419.96	4675.24	16561.95	5329.423	4610.379	-0.54	1.32	0.28	1.46
4-3	15.8	16412.05	5535.23	4755.07	16270.2	5479.935	4698.482	1.41	-0.23	0.93	1.70

4-4	17.7	31315.04	4268.02	4006.74	31063.4	4208.779	3962.666	0.10	2.17	0.89	2.35
5-1	38.5	15298.62	5332.44	4527.10	15153.49	5273.2	4473.245	-0.42	0.48	-0.19	0.67
5-2	38.7	28960.78	4026.74	3770.42	28566.61	3987.541	3731.981	-0.79	-0.21	0.10	0.83
5-3	38.8	14824.90	5497.96	4584.28	14705.38	5419.759	4524.794	-0.44	0.45	0.10	0.64
5-4	41.9	15321.37	5345.30	4559.79	15170.54	5286.401	4502.556	-0.79	1.45	2.26	2.80
5-5	41.9	28739.51	4048.38	3781.32	28387.22	4005.13	3740.178	1.70	1.01	2.17	2.94
5-6	42.6	14848.78	5442.48	4530.08	14768.83	5349.601	4467.254	-2.68	-0.92	1.22	3.09
6-1	41.8	9212.58	9108.53	5217.28	9136.696	8992.886	5152.727	0.00	-0.65	-0.46	0.80
6-2	44.1	9373.14	9037.43	5263.23	9274.065	8928.902	5190.471	0.00	-1.74	1.91	2.58
7-1	42.3	30110.71	4156.48	3904.93	29686.8	4122.006	3870.311	0.32	-0.05	0.00	0.33
7-2	42.6	15868.46	5419.09	4619.16	15738.41	5353.864	4565.626	-0.26	-0.39	0.01	0.47
7-3	44.4	29361.04	4214.40	3944.40	29028.64	4167.782	3901.192	1.08	2.60	0.00	2.82
7-4	45.6	15760.62	5403.29	4626.85	15675.78	5323.268	4563.386	-2.18	1.05	1.75	2.99
8-1	49.1	22555.24	4607.00	4546.00	22591.82	4557.302	4494.632	0.50	-0.13	-0.19	0.55
8-2	52.7	16786.82	5519.64	4834.44	16885.35	5395.378	4770.135	-0.10	2.31	1.36	2.69

Cartesian coordinates of all the conformers

All the structures are optimized at MP2/aug-cc-pVTZ. The relative energy (ΔE, in kcal/mol) includes the zero-point energy correction.

Isomer 1

Conformer 1-1
ΔE= 0.0

C	-1.381349000	-0.290051000	-0.069600000
C	0.034791000	0.010640000	0.362536000
N	0.918990000	-1.043265000	-0.066823000
O	0.337199000	1.303457000	-0.185665000
H	-1.710228000	-1.232699000	0.360117000
H	-1.419088000	-0.362974000	-1.156917000
H	-2.047715000	0.509640000	0.244156000
H	0.099593000	0.061200000	1.452588000
H	1.865342000	-0.894112000	0.260835000
H	0.943072000	-1.083641000	-1.079648000
H	1.217852000	1.554255000	0.114331000

Conformer 1-2
ΔE= 0.2

C	-1.335268000	-0.442164000	-0.088646000
C	0.037116000	0.009638000	0.366766000
N	1.031480000	-0.935344000	-0.043518000
O	0.356880000	1.301887000	-0.184741000
H	-1.579669000	-1.406320000	0.352333000
H	-1.346520000	-0.532835000	-1.175077000
H	-2.096950000	0.280497000	0.203081000
H	0.073363000	0.076152000	1.457675000
H	1.949856000	-0.615772000	0.240179000
H	1.037960000	-1.000602000	-1.055629000
H	-0.324530000	1.926343000	0.091268000

Conformer 1-3
ΔE=0.2

C	1.358891000	-0.351601000	0.074751000
C	-0.041645000	0.015587000	-0.371640000
N	-0.969989000	-1.001534000	0.053603000
O	-0.382602000	1.343990000	0.048539000
H	1.647574000	-1.318688000	-0.330894000
H	1.396198000	-0.413479000	1.165583000
H	2.067206000	0.406224000	-0.250840000
H	-0.091674000	0.076883000	-1.457931000

H	-1.919042000	-0.723900000	-0.165144000
H	-0.913091000	-1.146051000	1.055499000
H	-0.239914000	1.393917000	1.001534000

Conformer 1-4

ΔE= 0.4

C	-1.402025000	-0.155237000	-0.097139000
C	0.028423000	0.029602000	0.368524000
N	0.858796000	-1.023375000	-0.191124000
O	0.560996000	1.278295000	-0.039719000
H	-1.824180000	-1.081720000	0.294104000
H	-1.430078000	-0.190115000	-1.186349000
H	-2.009589000	0.677926000	0.248070000
H	0.060301000	0.075441000	1.460942000
H	0.431318000	-1.933105000	-0.068538000
H	1.764749000	-1.040441000	0.261929000
H	0.749550000	1.183095000	-0.982853000

Conformer 1-5

ΔE= 0.4

C	-1.367805000	-0.350438000	-0.096248000
C	0.027536000	0.020580000	0.362383000
N	1.091297000	-0.855433000	-0.098110000
O	0.272654000	1.326646000	-0.132100000
H	-1.640207000	-1.334805000	0.281617000
H	-1.397848000	-0.365137000	-1.185952000
H	-2.092736000	0.379351000	0.260354000
H	0.073524000	0.015501000	1.456744000
H	0.948964000	-1.097229000	-1.073065000
H	1.108459000	-1.716098000	0.435263000
H	1.221149000	1.472428000	-0.028204000

Conformer 1-6

ΔE= 3.3

C	-1.374271000	-0.307430000	-0.098309000
C	0.035058000	0.025090000	0.346848000
N	0.955699000	-0.953483000	-0.185017000
O	0.479886000	1.265798000	-0.194125000
H	-1.690066000	-1.270546000	0.304953000
H	-1.401241000	-0.345839000	-1.185389000
H	-2.072503000	0.451605000	0.253173000
H	0.069049000	0.097158000	1.443852000
H	0.688750000	-1.885695000	0.105437000
H	1.891731000	-0.774684000	0.158558000
H	0.020584000	1.970036000	0.276298000

Conformer 1-7
ΔE= 4.2

C	-1.157941000	-0.774596000	-0.108402000
C	0.043935000	0.030026000	0.367179000
N	1.326194000	-0.464338000	-0.078274000
O	-0.025942000	1.363068000	-0.126460000
H	-1.095593000	-1.799630000	0.256063000
H	-1.179155000	-0.787461000	-1.198505000
H	-2.092796000	-0.344193000	0.256540000
H	0.070409000	0.037433000	1.462158000
H	1.313825000	-0.598002000	-1.083705000
H	1.528595000	-1.357700000	0.352694000
H	-0.937066000	1.662794000	-0.038311000

Isomer 2

Conformer 2-1
1 0.0

N	-1.354054000	-0.563296000	0.120356000
C	-0.632315000	0.650102000	-0.278431000
C	0.777753000	0.557423000	0.274327000
O	1.410589000	-0.629889000	-0.169429000
H	-2.197129000	-0.685883000	-0.424914000
H	-1.636526000	-0.505213000	1.091860000
H	-1.103837000	1.579950000	0.056097000
H	-0.581227000	0.665345000	-1.366916000
H	0.742827000	0.590289000	1.370646000
H	1.379846000	1.397618000	-0.068687000
H	0.717088000	-1.305073000	-0.120529000

Conformer 2-2
ΔE= 1.3

N	-1.431314000	-0.533609000	0.247934000
C	-0.681088000	0.604439000	-0.278241000
C	0.719655000	0.576968000	0.281354000
O	1.346229000	-0.603881000	-0.228055000
H	-0.959259000	-1.387948000	-0.025457000
H	-2.358167000	-0.566973000	-0.157877000
H	-1.165778000	1.526566000	0.045363000
H	-0.612145000	0.624960000	-1.372155000
H	0.671289000	0.546902000	1.372311000
H	1.266896000	1.469418000	-0.033891000
H	2.175134000	-0.735051000	0.241931000

Conformer 2-3
ΔE=1.6

N	-1.527521000	-0.530010000	0.074667000

C	-0.681297000	0.598190000	-0.296734000
C	0.716874000	0.572693000	0.288683000
O	1.352570000	-0.608787000	-0.200117000
H	-1.681744000	-0.533692000	1.076575000
H	-1.039109000	-1.391544000	-0.141883000
H	-1.173596000	1.523621000	0.005890000
H	-0.596909000	0.615508000	-1.384271000
H	0.662328000	0.555991000	1.382246000
H	1.267439000	1.466593000	-0.020841000
H	2.220210000	-0.681409000	0.208863000

Conformer 2-4

ΔE= 1.9

N	1.531549000	-0.527583000	0.050484000
C	0.672728000	0.594076000	-0.312241000
C	-0.713966000	0.565724000	0.309858000
O	-1.426961000	-0.621059000	-0.039162000
H	1.014616000	-1.390729000	-0.077933000
H	1.759628000	-0.480862000	1.037024000
H	0.570639000	0.605995000	-1.400934000
H	1.171234000	1.524635000	-0.033564000
H	-1.278960000	1.458828000	0.024664000
H	-0.635989000	0.545746000	1.396922000
H	-1.658901000	-0.560859000	-0.971969000

Conformer 2-5

ΔE=1.8

N	1.447832000	-0.517063000	-0.230997000
C	0.673231000	0.603023000	0.292558000
C	-0.717309000	0.565603000	-0.304071000
O	-1.416017000	-0.625721000	0.064783000
H	0.944191000	-1.378286000	-0.051450000
H	2.342971000	-0.590590000	0.235256000
H	1.156029000	1.537081000	-0.001366000
H	0.584035000	0.610597000	1.388197000
H	-0.645644000	0.535721000	-1.389591000
H	-1.281764000	1.454620000	-0.011516000
H	-1.642038000	-0.555693000	0.998258000

Conformer 2-6

ΔE= 2.2

N	1.533073000	-0.501501000	0.068665000
C	0.671517000	0.625939000	-0.272810000
C	-0.740887000	0.563699000	0.285395000
O	-1.468444000	-0.563465000	-0.194087000
H	1.196300000	-1.357078000	-0.354257000

H	1.575016000	-0.644722000	1.070871000
H	0.618616000	0.686349000	-1.360096000
H	1.146707000	1.543003000	0.082270000
H	-1.305706000	1.438923000	-0.034744000
H	-0.709431000	0.563225000	1.380595000
H	-1.089245000	-1.349299000	0.211890000

Conformer 2-7
ΔE= 2.8

N	-1.888269000	-0.037051000	0.017777000
C	-0.542947000	0.525103000	-0.048211000
C	0.577470000	-0.503453000	0.024469000
O	1.869578000	0.084070000	-0.087823000
H	-2.010475000	-0.574072000	0.868224000
H	-2.044721000	-0.673444000	-0.754813000
H	-0.431328000	1.238853000	0.771647000
H	-0.444270000	1.089843000	-0.975200000
H	0.495889000	-1.197545000	-0.811997000
H	0.486886000	-1.084622000	0.948938000
H	2.002142000	0.657879000	0.673799000

Conformer 2-8
ΔE= 2.7

N	-1.887441000	0.055666000	-0.000017000
C	-0.549297000	-0.525116000	-0.000056000
C	0.577866000	0.492615000	0.000024000
O	1.807264000	-0.227082000	0.000058000
H	-2.024217000	0.643031000	-0.813689000
H	-2.024341000	0.642607000	0.813939000
H	-0.444894000	-1.164964000	-0.876236000
H	-0.444910000	-1.165074000	0.876046000
H	0.494450000	1.130144000	0.886820000
H	0.494525000	1.130202000	-0.886735000
H	2.531950000	0.406058000	-0.000303000

Conformer 2-9
ΔE= 2.7

N	-1.819227000	0.212922000	0.064421000
C	-0.548688000	-0.508802000	-0.010288000
C	0.587136000	0.487441000	0.073894000
O	1.803882000	-0.243467000	-0.061167000
H	-2.587269000	-0.433878000	0.189171000
H	-1.996177000	0.703784000	-0.803948000
H	-0.420161000	-1.107589000	-0.917569000
H	-0.483103000	-1.185060000	0.841402000
H	0.536782000	1.011596000	1.030520000

H	0.485662000	1.226720000	-0.728113000
H	2.537108000	0.369876000	0.045293000

Conformer 2-10

ΔE= 2.9

N	1.821662000	-0.184525000	0.101317000
C	0.543988000	0.508028000	-0.067812000
C	-0.586544000	-0.493225000	0.090600000
O	-1.864977000	0.096531000	-0.124263000
H	2.585248000	0.478413000	0.140901000
H	2.002561000	-0.783183000	-0.695924000
H	0.426377000	1.017775000	-1.029415000
H	0.463289000	1.263805000	0.716123000
H	-0.519440000	-0.967177000	1.072326000
H	-0.493815000	-1.275241000	-0.663419000
H	-2.040705000	0.696216000	0.607555000

Conformer 2-11

ΔE= 2.9

N	-1.821471000	-0.198277000	-0.045322000
C	-0.542976000	0.510149000	-0.022687000
C	0.587883000	-0.501903000	-0.056000000
O	1.868701000	0.119133000	-0.050474000
H	-2.588219000	0.448234000	-0.179111000
H	-1.985506000	-0.663823000	0.839276000
H	-0.410595000	1.164417000	0.849437000
H	-0.475963000	1.136763000	-0.911350000
H	0.531765000	-1.078461000	-0.976223000
H	0.486086000	-1.200005000	0.782249000
H	1.973681000	0.578270000	0.788890000

Conformer 2-12

ΔE= 4.2

N	-1.483417000	-0.557670000	0.142954000
C	-0.650308000	0.546609000	-0.328127000
C	0.715522000	0.511923000	0.321596000
O	1.453959000	-0.568178000	-0.236907000
H	-2.311489000	-0.651288000	-0.431066000
H	-1.803156000	-0.378900000	1.087364000
H	-1.086092000	1.537266000	-0.150283000
H	-0.510633000	0.436352000	-1.403792000
H	0.600044000	0.389270000	1.403879000
H	1.224705000	1.463150000	0.137140000
H	2.247581000	-0.697924000	0.290528000

Isomer 3

Conformer 3-1

ΔE= 0.0

C	-1.494576000	0.447741000	0.087982000
N	-0.620625000	-0.675869000	-0.220157000
C	0.706229000	-0.539013000	0.302361000
O	1.475108000	0.552688000	-0.218812000
H	-2.494232000	0.245258000	-0.290262000
H	-1.161108000	1.404009000	-0.330585000
H	-1.563085000	0.550406000	1.171621000
H	-0.576670000	-0.825537000	-1.220916000
H	1.278218000	-1.427003000	0.046619000
H	0.634086000	-0.456630000	1.389448000
H	1.156386000	1.366709000	0.183609000

Conformer 3-2

ΔE= 0.1

C	1.498772000	-0.455914000	0.094397000
N	0.621926000	0.660772000	-0.241115000
C	-0.698505000	0.544901000	0.298368000
O	-1.422594000	-0.641760000	-0.055323000
H	2.484254000	-0.277858000	-0.330666000
H	1.129337000	-1.424415000	-0.250646000
H	1.602766000	-0.499682000	1.178480000
H	0.590858000	0.809277000	-1.242121000
H	-1.267967000	1.430533000	0.012577000
H	-0.624292000	0.499628000	1.383451000
H	-1.689284000	-0.562723000	-0.977275000

Conformer 3-3

ΔE= 0.4

C	-1.519704000	-0.444773000	0.117045000
N	-0.557717000	0.517078000	-0.410921000
C	0.691594000	0.524538000	0.309974000
O	1.484081000	-0.589373000	-0.056087000
H	-2.432391000	-0.411358000	-0.473844000
H	-1.771023000	-0.265446000	1.169237000
H	-1.097233000	-1.443496000	0.034717000
H	-0.951157000	1.449104000	-0.431114000
H	0.568188000	0.428775000	1.394490000
H	1.202422000	1.465777000	0.090592000
H	1.481224000	-0.606508000	-1.021052000

Conformer 3-4

ΔE= 0.3

C	1.490808000	-0.458728000	0.115957000

N	0.622861000	0.660275000	-0.240006000
C	-0.694215000	0.553979000	0.285366000
O	-1.351817000	-0.623437000	-0.219454000
H	2.483379000	-0.283170000	-0.293451000
H	1.124802000	-1.426085000	-0.234358000
H	1.578104000	-0.496848000	1.201830000
H	0.579917000	0.770540000	-1.245619000
H	-1.260759000	1.442149000	-0.000226000
H	-0.627767000	0.498005000	1.375118000
H	-2.202725000	-0.710522000	0.224441000

Conformer 3-5
ΔE= 0.9

C	1.800156000	0.082630000	-0.063080000
N	0.470270000	-0.513147000	-0.006130000
C	-0.571634000	0.488109000	0.069934000
O	-1.841818000	-0.118493000	-0.001029000
H	2.549372000	-0.705709000	-0.062875000
H	2.015200000	0.765293000	0.768334000
H	1.900666000	0.641755000	-0.992782000
H	0.395589000	-1.116280000	0.805857000
H	-0.406071000	1.188738000	-0.755391000
H	-0.573160000	1.054981000	1.007696000
H	-1.810077000	-0.713238000	-0.760813000

Conformer 3-6
ΔE= 3.2

C	-1.506841000	-0.467185000	0.090070000
N	-0.588406000	0.563055000	-0.371777000
C	0.691105000	0.521675000	0.296034000
O	1.525970000	-0.501808000	-0.214734000
H	-2.484598000	-0.316856000	-0.361993000
H	-1.623462000	-0.487594000	1.182505000
H	-1.147654000	-1.443433000	-0.234153000
H	-0.996972000	1.481931000	-0.257944000
H	0.584154000	0.388320000	1.383804000
H	1.208422000	1.458617000	0.102742000
H	1.265604000	-1.334841000	0.188725000

Conformer 3-7
ΔE= 3.2

C	-1.500544000	-0.480486000	0.111596000
N	-0.590579000	0.548999000	-0.370900000
C	0.682083000	0.535045000	0.285624000
O	1.425962000	-0.552851000	-0.252624000
H	-2.457683000	-0.386789000	-0.396539000

H	-1.669301000	-0.432757000	1.195610000
H	-1.083588000	-1.455749000	-0.127609000
H	-1.003737000	1.468990000	-0.303275000
H	0.597293000	0.400794000	1.376262000
H	1.183357000	1.484211000	0.082012000
H	2.070777000	-0.826245000	0.407506000

Conformer 3-8

ΔE= 3.9

C	1.805713000	0.103170000	-0.010672000
N	0.488808000	-0.513996000	-0.087525000
C	-0.560002000	0.460783000	0.041434000
O	-1.788140000	-0.234387000	-0.090456000
H	2.568776000	-0.670636000	-0.048757000
H	1.963844000	0.702776000	0.895732000
H	1.945486000	0.754377000	-0.873286000
H	0.378240000	-1.204597000	0.646174000
H	-0.429715000	1.196369000	-0.758977000
H	-0.535392000	0.994025000	1.006303000
H	-2.482049000	0.317037000	0.284568000

Conformer 3-9

ΔE= 4.0

C	1.805764000	0.099712000	0.000666000
N	0.492278000	-0.517618000	-0.146460000
C	-0.561371000	0.451295000	0.085064000
O	-1.841564000	-0.093833000	-0.145587000
H	2.576741000	-0.659796000	-0.105937000
H	1.952309000	0.611830000	0.961095000
H	1.943633000	0.831994000	-0.794595000
H	0.399575000	-1.274685000	0.522517000
H	-0.430432000	1.255254000	-0.638657000
H	-0.512574000	0.888817000	1.094575000
H	-2.109045000	-0.585468000	0.636537000

Isomer 4

Conformer 4-1

ΔE= 0.0

C	-1.770185000	0.042026000	-0.000047000
O	-0.475712000	-0.528371000	-0.000081000
C	0.525002000	0.488615000	0.000071000
N	1.839440000	-0.063187000	0.000054000
H	-2.485220000	-0.775538000	-0.000145000
H	-1.931202000	0.660699000	-0.889014000
H	-1.931236000	0.660515000	0.889042000
H	0.396337000	1.114226000	0.889464000

H	0.396400000	1.114430000	-0.889188000
H	1.977850000	-0.644342000	-0.816630000
H	1.977781000	-0.644556000	0.816597000

Conformer 4-2
ΔE= 0.5

C	-1.475360000	0.438136000	0.091447000
O	-0.624053000	-0.590336000	-0.373342000
C	0.645069000	-0.577953000	0.272919000
N	1.523513000	0.509694000	-0.054394000
H	-2.445523000	0.285988000	-0.373031000
H	-1.108276000	1.430679000	-0.184229000
H	-1.586767000	0.392016000	1.180247000
H	1.113812000	-1.511146000	-0.028591000
H	0.495489000	-0.579561000	1.357167000
H	1.180596000	1.397782000	0.284298000
H	1.660248000	0.577973000	-1.054567000

Conformer 4-3
ΔE= 1.9

C	-1.468530000	-0.468358000	0.098279000
O	-0.642190000	0.591534000	-0.357582000
C	0.622357000	0.584230000	0.262015000
N	1.394123000	-0.581797000	-0.118003000
H	-2.418108000	-0.369798000	-0.419648000
H	-1.635170000	-0.386810000	1.177633000
H	-1.024429000	-1.438780000	-0.121315000
H	0.509612000	0.567348000	1.352192000
H	1.074594000	1.531533000	-0.035036000
H	2.090913000	-0.811319000	0.577017000
H	1.858283000	-0.447100000	-1.005926000

Conformer 4-4
ΔE= 3.8

C	-1.774161000	0.062357000	0.034785000
O	-0.495441000	-0.521607000	-0.112551000
C	0.516705000	0.454687000	0.029536000
N	1.784558000	-0.214958000	-0.052766000
H	-2.504890000	-0.732799000	-0.079527000
H	-1.952273000	0.826524000	-0.729220000
H	-1.892386000	0.522000000	1.021808000
H	0.346380000	0.986485000	0.978019000
H	0.452784000	1.183696000	-0.786562000
H	2.051383000	-0.624889000	0.831357000
H	2.515365000	0.414277000	-0.352028000

Isomer 5

Conformer 5-1

ΔE= 0.0

C	-1.511749000	-0.508985000	-0.104844000
C	-0.584230000	0.631180000	0.265041000
N	0.698379000	0.506389000	-0.423412000
O	1.432863000	-0.543362000	0.248251000
H	-2.475585000	-0.393795000	0.389564000
H	-1.083228000	-1.459887000	0.205546000
H	-1.671344000	-0.532272000	-1.181832000
H	-0.442157000	0.672409000	1.350219000
H	-1.002056000	1.586462000	-0.060353000
H	1.249136000	1.332544000	-0.207275000
H	1.649551000	-1.136458000	-0.479178000

Conformer 5-2

ΔE= 0.2

C	-1.841088000	-0.143770000	0.009530000
C	-0.478206000	0.525267000	-0.010764000
N	0.548682000	-0.501236000	0.139664000
O	1.821821000	0.133150000	-0.116590000
H	-2.629126000	0.602210000	-0.076100000
H	-1.984624000	-0.696393000	0.936200000
H	-1.948678000	-0.838721000	-0.824293000
H	-0.375503000	1.216264000	0.824988000
H	-0.334889000	1.093552000	-0.935303000
H	0.456429000	-1.142439000	-0.644899000
H	2.316810000	-0.079995000	0.681877000

Conformer 5-3

ΔE= 0.4

C	-1.474844000	-0.530469000	0.104825000
C	-0.591482000	0.640888000	-0.297751000
N	0.751219000	0.625677000	0.277619000
O	1.363666000	-0.629926000	-0.102453000
H	-2.452635000	-0.453768000	-0.370387000
H	-1.624926000	-0.545947000	1.185317000
H	-1.018479000	-1.471707000	-0.192788000
H	-1.033307000	1.587362000	0.022006000
H	-0.475077000	0.682501000	-1.380450000
H	0.654485000	0.527989000	1.286105000
H	2.180038000	-0.329275000	-0.515955000

Conformer 5-4

ΔE= 3.5

C	-1.519637000	-0.482463000	-0.119046000

C	-0.559541000	0.614353000	0.298913000
N	0.700450000	0.508493000	-0.425351000
O	1.430426000	-0.629789000	0.041805000
H	-2.460045000	-0.394347000	0.423649000
H	-1.091036000	-1.460829000	0.086525000
H	-1.719835000	-0.414131000	-1.186562000
H	-0.400600000	0.587208000	1.388257000
H	-0.966314000	1.597535000	0.052863000
H	1.281902000	1.313754000	-0.208324000
H	1.484438000	-0.541673000	1.007404000

Conformer 5-5

ΔE= 3.5

C	1.836330000	-0.129864000	0.014357000
C	0.462141000	0.514758000	-0.020069000
N	-0.549192000	-0.525988000	-0.128181000
O	-1.852751000	0.057731000	-0.066119000
H	2.610808000	0.632561000	0.074738000
H	1.998482000	-0.722846000	-0.883319000
H	1.941727000	-0.782677000	0.881580000
H	0.356236000	1.159059000	-0.891702000
H	0.311104000	1.137357000	0.875109000
H	-0.480883000	-1.137877000	0.682667000
H	-1.861954000	0.625133000	0.721419000

Conformer 5-6

ΔE= 4.3

C	-1.471665000	-0.514458000	0.112426000
C	-0.574183000	0.654152000	-0.281708000
N	0.781106000	0.628408000	0.255655000
O	1.469654000	-0.546592000	-0.183883000
H	-2.475851000	-0.369220000	-0.284569000
H	-1.548834000	-0.598946000	1.197632000
H	-1.109003000	-1.462006000	-0.286970000
H	-1.007519000	1.592356000	0.070336000
H	-0.484089000	0.719085000	-1.365617000
H	0.738657000	0.560078000	1.270674000
H	0.936754000	-1.305631000	0.095688000

Isomer 6

Conformer 6-1

ΔE= 0.0

C	1.193916000	-0.644397000	0.071053000
N	0.000001000	0.018572000	-0.431590000
C	-1.193904000	-0.644416000	0.071049000
O	-0.000020000	1.323440000	0.200648000

H	1.211835000	-1.657332000	-0.328711000
H	1.202655000	-0.683450000	1.166596000
H	2.073605000	-0.109121000	-0.275838000
H	-2.073603000	-0.109183000	-0.275880000
H	-1.202663000	-0.683428000	1.166593000
H	-1.211780000	-1.657368000	-0.328674000
H	0.000033000	1.915235000	-0.560755000

Conformer 6-2
ΔE= 2.3

C	1.197018000	-0.625368000	0.081683000
N	-0.000002000	0.002320000	-0.444970000
C	-1.197018000	-0.625366000	0.081694000
O	0.000001000	1.362126000	-0.011347000
H	1.226379000	-1.659154000	-0.259005000
H	1.211419000	-0.613913000	1.183544000
H	2.068264000	-0.096410000	-0.293881000
H	-1.226386000	-1.659151000	-0.258996000
H	-2.068267000	-0.096404000	-0.293859000
H	-1.211408000	-0.613914000	1.183555000
H	0.000007000	1.330104000	0.963952000

Isomer 7
Conformer 7-1
ΔE= 0.0

C	-1.828170000	-0.176402000	0.000000000
C	-0.489969000	0.525069000	-0.000008000
O	0.507741000	-0.492797000	0.000016000
N	1.793411000	0.153844000	-0.000016000
H	-2.631652000	0.559050000	-0.000012000
H	-1.931620000	-0.803112000	0.883868000
H	-1.931616000	-0.803138000	-0.883849000
H	-0.367445000	1.154457000	0.884849000
H	-0.367429000	1.154423000	-0.884885000
H	2.261406000	-0.244188000	-0.810085000
H	2.261385000	-0.244028000	0.810146000

Conformer 7-2
ΔE= 0.4

C	1.496490000	-0.536750000	-0.066047000
C	0.595490000	0.640386000	0.241875000
O	-0.670368000	0.545655000	-0.410188000
N	-1.414073000	-0.512326000	0.222108000
H	2.464625000	-0.405430000	0.418032000
H	1.654979000	-0.622359000	-1.140224000
H	1.052694000	-1.459329000	0.302573000

H	1.018414000	1.569342000	-0.140955000
H	0.437710000	0.741237000	1.318073000
H	-1.619992000	-1.156523000	-0.537143000
H	-2.298852000	-0.067704000	0.451427000

Conformer 7-3

ΔE= 2.1

C	-1.821281000	-0.132478000	0.000156000
C	-0.450372000	0.502147000	0.000119000
O	0.506719000	-0.550364000	-0.000129000
N	1.836177000	-0.047178000	-0.000186000
H	-2.590137000	0.638970000	0.000346000
H	-1.949866000	-0.753850000	0.883960000
H	-1.950036000	-0.753587000	-0.883808000
H	-0.303567000	1.129358000	0.887861000
H	-0.303736000	1.129621000	-0.887466000
H	1.910215000	0.562197000	0.814944000
H	1.910057000	0.562431000	-0.815156000

Conformer 7-4

ΔE= 3.4

C	-1.495321000	0.502980000	-0.090842000
C	-0.551700000	-0.626374000	0.277690000
O	0.698690000	-0.569977000	-0.406760000
N	1.522217000	0.494169000	0.051797000
H	-2.460155000	0.348357000	0.392095000
H	-1.646344000	0.528952000	-1.168630000
H	-1.122722000	1.473284000	0.235217000
H	-0.973344000	-1.586135000	-0.019884000
H	-0.372203000	-0.651389000	1.359333000
H	1.611680000	0.371117000	1.060720000
H	1.000176000	1.356809000	-0.088431000

Isomer 8

Conformer 8-1

ΔE= 0.0

C	-1.654785000	-0.350062000	-0.019803000
N	-0.520543000	0.517569000	-0.310985000
O	0.536504000	0.117746000	0.582108000
C	1.664441000	-0.215027000	-0.213246000
H	-2.510827000	0.024792000	-0.580707000
H	-1.899654000	-0.383545000	1.044357000
H	-1.425510000	-1.356014000	-0.363399000
H	-0.745474000	1.453728000	0.016070000
H	2.016584000	0.650185000	-0.776227000
H	1.426590000	-1.022603000	-0.907310000

H	2.432122000	-0.540962000	0.485540000

Conformer 8-2

ΔE= 3.7

C	1.477509000	0.464376000	-0.103169000
N	0.626879000	-0.664831000	0.249638000
O	-0.634423000	-0.572757000	-0.411140000
C	-1.451312000	0.458250000	0.124717000
H	2.420644000	0.328775000	0.425036000
H	1.074499000	1.447794000	0.153317000
H	1.669023000	0.423893000	-1.172489000
H	0.415969000	-0.638776000	1.246798000
H	-1.038682000	1.453210000	-0.050141000
H	-1.605430000	0.310841000	1.198606000
H	-2.405966000	0.374377000	-0.388761000
